# Multicriteria Decision-Making Approach for Aggregation Operators of Pythagorean Fuzzy Hypersoft Sets

**DOI:** 10.1155/2021/2036506

**Published:** 2021-09-22

**Authors:** Imran Siddique, Rana Muhammad Zulqarnain, Rifaqat Ali, Fahd Jarad, Aiyared Iampan

**Affiliations:** ^1^Department of Mathematics, School of Science, University of Management and Technology, Lahore 54770, Pakistan; ^2^Department of Mathematics, School of Science, University of Management and Technology, Sialkot Campus, Lahore, Pakistan; ^3^Department of Mathematics, College of Science and Arts, King Khalid University, Muhayil, Abha 61413, Saudi Arabia; ^4^Department of Mathematics, Cankaya University, Etimesgut, Ankara, Turkey; ^5^Department of Medical Research, China Medical University Hospital, China Medical University, Taichung, Taiwan; ^6^Department of Mathematics, School of Science, University of Phayao, Mae Ka, Mueang, Phayao 56000, Thailand

## Abstract

The Pythagorean fuzzy hypersoft set (PFHSS) is the most advanced extension of the intuitionistic fuzzy hypersoft set (IFHSS) and a suitable extension of the Pythagorean fuzzy soft set. In it, we discuss the parameterized family that contracts with the multi-subattributes of the parameters. The PFHSS is used to correctly assess insufficiencies, anxiety, and hesitancy in decision-making (DM). It is the most substantial notion for relating fuzzy data in the DM procedure, which can accommodate more uncertainty compared to available techniques considering membership and nonmembership values of each subattribute of given parameters. In this paper, we will present the operational laws for Pythagorean fuzzy hypersoft numbers (PFHSNs) and also some fundamental properties such as idempotency, boundedness, shift-invariance, and homogeneity for Pythagorean fuzzy hypersoft weighted average (PFHSWA) and Pythagorean fuzzy hypersoft weighted geometric (PFHSWG) operators. Furthermore, a novel multicriteria decision-making (MCDM) approach has been established utilizing presented aggregation operators (AOs) to resolve decision-making complications. To validate the useability and pragmatism of the settled technique, a brief comparative analysis has been conducted with some existing approaches.

## 1. Introduction

Decision-making (DM) is one of the enormously charming apprehensions these days, to pick a proper alternate for any precise intention. It is pretended that facts about probable selections are gathered in crisp numbers, but in real cases, aggregated statistics mostly suppress misinformation. The decision-maker needs to re-evaluate the choices prospering by the several indicative stipulations such as intervals and numbers. However, in quite a lot of instances, it is difficult for one person to take action because of numerous feedback loops in the record. One reason is lack of expertise or paradox. Hence, a chain of assertions had been proposed to contemplate the measuring along with the scientific method of the specified negative aspects. Zadeh was the first mathematician who developed the notion of fuzzy sets (FSs) [1] to address vague and imprecise information. In general, we need to keep a watch on membership (MD) as a nonmembership degree (NMD) but FS deals only with the MD. To overcome this problem, Atanossov [2] defined for the first time a new set known as intuitionistic fuzzy set (IFSs), which deals with the MD and NMD both at the same time. Surely, IFS is the extension of the FS, and also, it deals with more information compared to FS. Although IFS was a new domain for work, there were limitations to it. IFSs are unable to handle data that is irreconcilable and inexact. The theories presented above were fairly suggested by experts, and the sum of two MD and NMD cannot exceed one since the preceding effort is thought to anticipate the environment among MD and NMD. If the experts estimated MD and NMD to be 0.4 and 0.7, then 0.4 + 0.7 ≥ 1, and IFSs would be unable to manage the issue. By improving MD+NMD ≤ 1 to MD^2^+NMD^2^ ≤ 1, Yager [3, 4] extended the idea of IFSs to Pythagorean fuzzy sets (PFSs) to overcome the abovementioned issues. To overcome the MCDM challenge, Zhang and Xu [5] designed operating guidelines for PFSs and built up the DM approach. Wang and Li [6] proposed some unique operational laws and AOs for PFSs that took into account their desirable features' interactions. Gao et al. [7] developed the concept of PFSs and constructed some AOs that take into account the interaction. They also provided a method for multiattribute decision-making (MADM) based on their existing operators.

Wei [8] created aggregation operators (AO) for PFS based on well-established operational laws. Talukdar et al. [9] used linguistic PFSs to make medical diagnoses and introduced certain distance and accuracy functions. Wang et al. [10] extended the concept of PFSs by proposing interactive Hamacher AOs and a MADM approach to handle DM problems. Ejegwa et al. [11] proposed an MCDM technique and produced a correlation metric for IFSs. Peng and Yang [12] listed some fundamental PFS operations as well as their basic characteristics. Based on his derived logarithmic operational principles, Garg [13] offered various AOs for PFSs. Based on their developed operational regulations, Arora and Garg [14] introduced prioritized AOs for linguistic IFSs. Ma and Xu [15] proposed new AOs for PFSs and provided PFN comparison laws.

The abovementioned ideas and DM approaches are applied in a variety of domains, including medical diagnosis, artificial intelligence, and economics. However, due to their inability to use the parameterization tool, these models have some limitations. Molodtsov [16] offered the concept of soft sets (SSs) to address the aforementioned problems when considering substitution parameterization. Maji et al. [17] constructed a DM approach to tackle DM challenges using their produced operations and extended the idea of SSs with multiple necessary operators and their appropriate assets. Garg and Arora [18] provided a generalized form of IFSSs with AOs and a DM approach to handle DM challenges based on their created AOs. The correlation coefficient (CC) and the weighted correlation coefficient (WCC) for IFSSs were developed by Garg and Arora [19]. They also demonstrated how to use the TOPSIS methodology to find MADM issues using their established correlation metrics. Zulqarnain et al. [20] expanded on interval-valued IFSSs and proposed AOs for them. They also presented the CC and WCC for interval-valued IFSSs as well as the TOPSIS technique for resolving MADM problems, based on the correlation measures they offered.

Peng et al. [21] developed the PFSSs' hypothesis by combining two existing ideas, PFSs and SSs. Athira et al. [22] expanded on the concept of PFSSs by introducing new distance metrics and developing a DM technique. The operating laws for Pythagorean fuzzy soft numbers (PFSNs) were advanced by Zulqarnain et al. [23], and the AOs for PFSNs were planned. They also proposed a MADM strategy for dealing with these DM worries based on their existing AOs. Riaz et al. [24] defined *m* polar PFSSs and proposed the TOPSIS approach for resolving multiple criteria group decision-making (MCGDM) problems. In light of the interaction, Zulqarnain et al. [25] developed AOs for PFSSs and devised a decision-making approach based on their AOs. Riaz et al. [26] introduced PFSS similarity measurements and underlined their critical importance. Zulqarnain et al. [27] developed the TOPSIS approach based on the CC and expanded the impression of PFSSs. They also presented an MCGDM approach for supplier selection, which they created themselves.

Current research is not able to confirm the situation wherever some criterion of a set of attributes has subattributes. Samarandche [28] progressed the idea of the hypersoft set (HSS), which permeates the parameter function *f* with multiple subattributes, which is a feature of Cartesian products with *n* attributes. The Samarandche HSS is the most suitable theory comparative to SS and other existing notions. It can handle uncertain and imprecise information considering the multi-subattributes of the considered parameters. Several extensions of HSS with their decision-making approaches have been presented. Zulqarnain et al. [29] extended the notion of neutrosophic HSS (NHSS) with their necessary properties. Zulqarnain et al. [30] extended the PFSS and presented the idea of PFHSS with its basic operations and properties. They also developed the CC for PFHSS and offered a decision-making methodology based on their developed CC. Samad et al. [31] prolonged the notion of PFHSS and established the TOPSIS approach for PFHSS utilizing correlation measures for PFHSS. They utilized their developed TOPSIS approach to resolve MCDM complications. Zulqarnain et al. [32] prolonged the NHSS to neutrosophic hypersoft matrices with some basic operations such as necessity, possibility operations, and logical operations and discussed their desirable properties. They also proposed a MADM technique to resolve decision-making difficulties. Zulqarnain et al. [33, 34] established the CC, WCC, and AOs for IFHSSs and established the TOPSIS method to solve MADM problems based on their developed correlation measures. Zulqarnain et al. [35] established the TOPSIS approach for PFHSSs utilizing the CC and WCC. The above-presented are compatible only for MD and NMD of the multi-subattributes. These theories are unable to handle the circumstances whenever the experts considered the MD = 0.7 and NDM = 0.6. To overcome such types of difficulties, we need to develop operational laws for PFHSNs and AOs for PFHSSs based on presented operational laws. The core objective of the following research is to develop two novel AOs such as PFHSWA and PFHSWG operators. Furthermore, using the established operators, an MCDM technique has been offered.

The organization of the following paper is given as follows: in Section 2, we discuss some fundamental concepts which help us to develop the structure of the following article. In Section 3, we proposed novel operational laws for PFHSS and utilized the developed operational laws to establish PFHSWA and PFHSWG operators. A DM technique has been organized to solve MCDM problems based on offered AOs in Section 4. Furthermore, a comprehensive comparative discussion has been presented to ensure the validity and pragmatism of the proposed MCDM approach in Section 5.

## 2. Preliminaries

In this section, we remember some fundamental notions such as SS, HSS, IFHSS, and PFHSS.

**Definition 1 (see [16]).**

Let *𝒰* and *ε* be the universe of discourse and set of attributes, respectively. Let *𝒫*(*𝒰*) be the power set of *𝒰* and *𝒜*⊆*ε*. A pair (ℱ, *𝒜*) is called a SS over *𝒰*, and its mapping expressed as follows:

(1) $$\begin{array}{c} ℱ:A\;⟶P\left(U\right). \end{array}$$

Also, it can be defined as follows:

(2) $$\begin{array}{c} \left(ℱ,A\right)=\left\{ℱ\left(e\right)\in P\left(U\right):e\in \varepsilon ,ℱ\left(e\right)=\emptyset \text{ if }e\notin A\right\}. \end{array}$$

**Definition 2 (see [28]).**

Let *𝒰* be a universe of discourse and *𝒫*(*𝒰*) be a power set of *𝒰*, and *k* = {*k*_1_, *k*_2_, *k*_3_,..., *k*_*n*_}, (*n* ≥ 1), and *K*_*i*_ represented the set of attributes and their corresponding subattributes such as *K*_*i*_∩*K*_*j*_=*φ*, where *i* ≠ *j* for each *n* ≥ 1 and *i* and *j* ∈ {1,2,3,…*n*}. Assume that $K_{1}\times K_{2}\times K_{3}\times \ldots \times K_{n}=\overset{\ldots }{𝒜}=\left\{d_{1h}\times d_{2k}\times \ldots \times d_{nl}\right\}$ is a collection of subattributes, where 1 ≤ *h* ≤ *α*, 1 ≤ *k* ≤ *β*, and 1 ≤ *l* ≤ *γ*, and *α*, *β*, and *γ*∈ ℕ. Then, the pair (ℱ, *K*_1_ × *K*_2_ × *K*_3_ × … × *K*_*n*_ = (ℱ, $\overset{\ldots }{𝒜}$) is known as HSS, defined as follows:

(3) $$\begin{array}{c} ℱ:K_{1}\times K_{2}\times K_{3}\times \ldots \times K_{n}=\overset{\ldots }{A}⟶\;P\left(U\right). \end{array}$$

It is also defined as

(4) $$\begin{array}{c} \left(ℱ,\overset{\ldots }{A}\;\right)=\left\{\overset{ˇ}{d},\;{ℱ}_{\overset{\ldots }{A}}\left(\overset{ˇ}{d}\right):\;\overset{ˇ}{d}\in \overset{\ldots }{A},\;d{ℱ}_{\overset{\ldots }{A}}\left(\overset{ˇ}{d}\right)\;\in \;P\left(U\right)\right\}. \end{array}$$

**Definition 3 (see [28]).**

Let *𝒰* be a universe of discourse and *𝒫*(*𝒰*) be a power set of *𝒰*, and *k* = {*k*_1_, *k*_2_, *k*_3_,..., *k*_*n*_}, (*n* ≥ 1), and *K*_*i*_ represented the set of attributes and their corresponding subattributes such as *K*_*i*_∩*K*_*j*_=*φ*, where *i* ≠ *j* for each *n* ≥ 1 and *i* and *j* ∈ {1,2,3,…*n*}. Assume that $K_{1}\times K_{2}\times K_{3}\times \ldots \times K_{n}=\overset{\ldots }{𝒜}=\left\{d_{1h}\times d_{2k}\times \ldots \times d_{nl}\right\}$ is a collection of subattributes, where 1 ≤ *h* ≤ *α*, 1 ≤ *k* ≤ *β*, and 1 ≤  *l* ≤ *γ* and *α*, *β*, and *γ*∈ ℕ, and IFS^*𝒰*^ be a collection of all fuzzy subsets over *𝒰*. Then, the pair ($ℱ,K_{1}\times K_{2}\times K_{3}\times \ldots \times K_{n}=\left(ℱ,\overset{\ldots }{𝒜}\right)$ is known as IFHSS, defined as follows:

(5) $$\begin{array}{c} ℱ:K_{1}\times K_{2}\times K_{3}\times \ldots \times K_{n}=\overset{\ldots }{A}⟶\;{\text{IFS}}^{U}. \end{array}$$

It is also defined as

(6) $$\begin{array}{c} \left(ℱ,\overset{\ldots }{A}\;\right)=\left\{\left(\overset{ˇ}{d},\;{ℱ}_{\overset{\ldots }{A}}\left(\overset{ˇ}{d}\right)\right):\;\overset{ˇ}{d}\in \overset{\ldots }{A},\;{ℱ}_{\overset{\ldots }{A}}\left(\overset{ˇ}{d}\right)\;\in \;{\text{IFS}}^{U}\in \;\left[0,\;1\right]\right\}, \end{array}$$

where

(7) $$\begin{array}{c} {ℱ}_{\overset{\ldots }{A}}\left(d\overset{ˇ}{d}\right)=\left\{\delta ,\;T_{ℱ\left(\overset{ˇ}{d}\right)}\left(\delta \right),\;J_{ℱ\left(\overset{ˇ}{d}\right)}\left(\delta \right):\;\delta \in U\right\}, \end{array}$$

where ${𝒯}_{ℱ\left(\overset{ˇ}{d}\right)}\left(\delta \right)$ and ${𝒥}_{ℱ\left(\overset{ˇ}{d}\right)}\left(\delta \right)$ signify the Mem and NMem values of the attributes: ${𝒯}_{ℱ\left(\overset{ˇ}{d}\right)}\left(\delta \right)$, ${𝒥}_{ℱ\left(\overset{ˇ}{d}\right)}\left(\delta \right)\in \;\left[0,\;1\right]$, and $0\leq {𝒯}_{ℱ\left(\overset{ˇ}{d}\right)}\left(\delta \right)+{𝒥}_{ℱ\left(\overset{ˇ}{d}\right)}\left(\delta \right)\leq 1$.

Whenever the sum of MD and NMD of the multi-subattributes of the alternatives exceeded one, then the above-defined IFHSS is unable to handle the circumstances. To handle this scenario, Zulqarnain et al. [34] developed the PFHSS given as follows.

**Definition 4 (see [34]).**

Let *𝒰* be a universe of discourse and *𝒫*(*𝒰*) be a power set of *𝒰*, and *k* = {*k*_1_, *k*_2_, *k*_3_,..., *k*_*n*_}, (*n* ≥ 1), and *K*_*i*_ represented the set of attributes and their corresponding subattributes such as *K*_*i*_∩*K*_*j*_=*φ*, where *i* ≠ *j* for each *n* ≥ 1 and *i* and *j* ∈ {1,2,3,…*n*}. Assume that $K_{1}\times K_{2}\times K_{3}\times \ldots \times K_{n}=\overset{\ldots }{𝒜}=\left\{d_{1h}\times d_{2k}\times \ldots \times d_{nl}\right\}$ is a collection of subattributes, where 1 ≤ *h* ≤ *α*, 1 ≤ *k* ≤ *β*, and 1 ≤ *l* ≤ *γ* and *α*, *β*, and *γ*∈ ℕ and PFS^*𝒰*^ be a collection of all fuzzy subsets over *𝒰*. Then, the pair ($ℱ,K_{1}\times K_{2}\times K_{3}\times \ldots \times K_{n}=\left(ℱ,\overset{\ldots }{𝒜}\right)$ is known as PFHSS, defined as follows:

(8) $$\begin{array}{c} ℱ:K_{1}\times K_{2}\times K_{3}\times \ldots \times K_{n}=\overset{\ldots }{A}⟶\;{\text{PFS}}^{U}. \end{array}$$

It is also defined as

(9) $$\begin{array}{c} \left(ℱ,\overset{\ldots }{A}\right)=\left\{\left(\overset{ˇ}{d},\;{ℱ}_{\overset{\ldots }{A}}\left(\overset{ˇ}{d}\right)\right):\overset{ˇ}{d}\in \overset{\ldots }{A},{ℱ}_{\overset{\ldots }{A}}\left(\overset{ˇ}{d}\right)\in {\text{PFS}}^{U}\in \left[0,1\right]\right\}, \end{array}$$

where

(10) $$\begin{array}{c} {ℱ}_{\overset{\ldots }{A}}\left(\overset{ˇ}{d}\right)=\left\{\langle \delta ,\;T_{ℱ\left(\overset{ˇ}{d}\right)}\left(\delta \right),\;J_{ℱ\left(\overset{ˇ}{d}\right)}\left(\delta \right)\rangle :\;\delta \in U\right\}, \end{array}$$

where ${𝒯}_{ℱ\left(\overset{ˇ}{d}\right)}\left(\delta \right)$ and ${𝒥}_{ℱ\left(\overset{ˇ}{d}\right)}\left(\delta \right)$ signify the Mem and NMem values of the attributes: ${𝒯}_{ℱ\left(\overset{ˇ}{d}\right)}\left(\delta \right)$, ${𝒥}_{ℱ\left(\overset{ˇ}{d}\right)}\left(\delta \right)$∈ [0,  1], and $0\leq {\left({𝒯}_{ℱ\left(\overset{ˇ}{d}\right)}\left(\delta \right)\right)}^{2}+{\left({𝒥}_{ℱ\left(\overset{ˇ}{d}\right)}\left(\delta \right)\right)}^{2}\leq 1$.

A Pythagorean fuzzy hypersoft number (PFHSN) can be stated as $ℱ=\left\{\left({𝒯}_{ℱ\left(\overset{ˇ}{d}\right)}\left(\delta \right),{𝒥}_{ℱ\left(\overset{ˇ}{d}\right)}\left(\delta \right)\right)\right\}$, where $0\leq {\left({𝒯}_{ℱ\left(\overset{ˇ}{d}\right)}\left(\delta \right)\right)}^{2}+{\left({𝒥}_{ℱ\left(\overset{ˇ}{d}\right)}\left(\delta \right)\right)}^{2}\leq 1$.

**Remark 1 .**

If ${\left({𝒯}_{ℱ\left(\overset{ˇ}{d}\right)}\left(\delta \right)\right)}^{2}+{\left({𝒥}_{ℱ\left(\overset{ˇ}{d}\right)}\left(\delta \right)\right)}^{2}$ and $\;{𝒯}_{ℱ\left(\overset{ˇ}{d}\right)}\left(\delta \right)+{𝒥}_{ℱ\left(\overset{ˇ}{d}\right)}\left(\delta \right)\leq 1$ both hold, then PFHSS was reduced to IFHSS [33].

For readers' suitability, the PFHSN ${ℱ}_{\delta_{i}}\left(\overset{ˇ}{d_{j}}\right)=\left\{\left({𝒯}_{ℱ\left(\overset{ˇ}{d_{j}}\right)}\left(\delta_{i}\right),{𝒥}_{ℱ\left(\overset{ˇ}{d_{j}}\right)}\left(\delta_{i}\right)\right)|,\delta_{i}\in 𝒰\right\}$ can be written as ${𝔍}_{{\overset{ˇ}{d}}_{ij}}=\langle {𝒯}_{ℱ\left({\overset{ˇ}{d}}_{ij}\right)},\;{𝒥}_{ℱ\left({\overset{ˇ}{d}}_{ij}\right)}\rangle$. The score function for ${𝔍}_{\overset{ˇ}{d_{ij}}}$ is expressed as follows:

(11) $$\begin{array}{c} S\left(J_{\overset{ˇ}{d_{ij}}}\right)=T_{ℱ{\left(\overset{ˇ}{d_{ij}}\right)}^{2}}-J_{ℱ{\left(\overset{ˇ}{d_{ij}}\right)}^{2}},\;S\left(J_{\overset{ˇ}{d_{ij}}}\right)\in \left[-1,\;1\right]. \end{array}$$

But, sometimes, the scoring function such as ${𝔍}_{\overset{ˇ}{d_{11}}}=\langle 0.4,0.7\rangle$ and ${𝔍}_{\overset{ˇ}{d_{12}}}=\langle 0.5,\;0.8\rangle$ cannot provide suitable outcomes to compute the PFHSNs. It is difficult to conclude which alternative is more suitable $𝕊\left({𝔍}_{\overset{ˇ}{d_{11}}}\right)=0.3=𝕊\left({𝔍}_{\overset{ˇ}{d_{12}}}\right)$. To intimidate such complications, the accuracy function had been developed:

(12) $$\begin{array}{c} H\left(J_{\overset{ˇ}{d_{ij}}}\right)=T_{ℱ{\left(\overset{ˇ}{d_{ij}}\right)}^{2}}+J_{ℱ{\left(\overset{ˇ}{d_{ij}}\right)}^{2}},\;H\left(J_{\overset{ˇ}{d_{ij}}}\right)\in \left[0,\;1\right]. \end{array}$$

The following comparison laws have been projected to compute two PFHSNs ${𝔍}_{\overset{ˇ}{d_{ij}}}$ and ${𝔗}_{\overset{ˇ}{d_{ij}}}$:

(1) If $𝕊\left({𝔍}_{\overset{ˇ}{d_{ij}}}\right)>𝕊\left({𝔗}_{\overset{ˇ}{d_{ij}}}\right)$, then ${𝔍}_{\overset{ˇ}{d_{ij}}}>{𝔗}_{\overset{ˇ}{d_{ij}}}$
(2) If $𝕊\left({𝔍}_{\overset{ˇ}{d_{ij}}}\right)=𝕊\left({𝔗}_{\overset{ˇ}{d_{ij}}}\right)$, then
  - If $H\left({𝔍}_{\overset{ˇ}{d_{ij}}}\right)>H\left({𝔗}_{\overset{ˇ}{d_{ij}}}\right)$, then ${𝔍}_{\overset{ˇ}{d_{ij}}}>{𝔗}_{\overset{ˇ}{d_{ij}}}$
  - If $H\left({𝔍}_{\overset{ˇ}{d_{ij}}}\right)=H\left({𝔗}_{\overset{ˇ}{d_{ij}}}\right)$, then ${𝔍}_{\overset{ˇ}{d_{ij}}}={𝔗}_{\overset{ˇ}{d_{ij}}}$

## 3. Aggregation Operators for Pythagorean Fuzzy Hypersoft Numbers

In the following section, we will prove some fundamental properties for PFHSWA and PFHSWG operators such as idempotency, boundedness, shift-invariance, and homogeneity.

### 3.1. Operational Laws for PFHSNs

**Definition 5 .**

Let ${𝔍}_{\overset{ˇ}{d_{k}}}=\left({𝒯}_{\overset{ˇ}{d_{k}}},\;{𝒥}_{\overset{ˇ}{d_{k}}}\right)$, ${𝔍}_{\overset{ˇ}{d_{11}}}=\left({𝒯}_{\overset{ˇ}{d_{11}}},\;{𝒥}_{\overset{ˇ}{d_{11}}}\right)$, and ${𝔍}_{\overset{ˇ}{d_{12}}}=\left({𝒯}_{\overset{ˇ}{d_{12}}},\;{𝒥}_{\overset{ˇ}{d_{12}}}\right)$ represent the PFHSNs and *α* is a positive real number. Then, operational laws for PFHSNs can be expressed as follows:

1. ${𝔍}_{\overset{ˇ}{d_{11}}}⊕{𝔍}_{\overset{ˇ}{d_{12}}}=\langle \sqrt{{𝒯}_{\overset{ˇ}{d_{11}^{2}}}+{𝒯}_{\overset{ˇ}{d_{12}^{2}}}-{𝒯}_{\overset{ˇ}{d_{11}^{2}}}{𝒯}_{\overset{ˇ}{d_{12}^{2}}}},\;{𝒥}_{\overset{ˇ}{d_{11}}}{𝒥}_{\overset{ˇ}{d_{12}}}\rangle$
2. ${𝔍}_{\overset{ˇ}{d_{11}}}⊗{𝔍}_{\overset{ˇ}{d_{12}}}=\langle {𝒯}_{\overset{ˇ}{d_{11}}}{𝒯}_{\overset{ˇ}{d_{12}}},\;\sqrt{{𝒥}_{d_{11}^{2}}+{𝒥}_{\overset{ˇ}{d_{12}^{2}}}-{𝒥}_{d_{11}^{2}}{𝒥}_{\overset{ˇ}{d_{12}^{2}}}}\rangle$
3. $\alpha {𝔍}_{\overset{ˇ}{d_{k}}}=\langle \sqrt{1-{\left(1-{𝒯}_{\overset{ˇ}{d_{k}^{2}}}\right)}^{\alpha }},\;{𝒥}_{\overset{ˇ}{d_{k}^{\alpha }}}\rangle$
4. ${𝔍}_{\overset{ˇ}{d_{k}}}^{\alpha }=\langle {𝒯}_{\overset{ˇ}{d_{k}}}^{\alpha },\;\sqrt{1-{\left(1-{𝒥}_{\overset{ˇ}{d_{k}}}^{2}\right)}^{\alpha }}\rangle$

In the following, we will describe some AOs for PFHSNs using the above-presented operational laws.

**Definition 6 .**

Let ${𝔍}_{\overset{ˇ}{d_{ij}}}=\left({𝒯}_{\overset{ˇ}{d_{ij}}},\;{𝒥}_{\overset{ˇ}{d_{ij}}}\right)$ be a PFHSN, Ω_*i*_ and *γ*_*j*_ be weight vectors for experts and multi-subattributes of the considered attributes consistently under definite surroundings Ω_*i*_ > 0, ∑_*i*=1_^*n*^Ω_*i*_=1, *γ*_*j*_ > 0, and ∑_*j*=1_^*m*^*γ*_*j*_=1. Then, PFHSWA: Δ^*n*^⟶Δ defined as follows:

(13) $$\begin{array}{c} \text{PFHSWA }\left(J_{\overset{ˇ}{d_{11}}},\;J_{\overset{ˇ}{d_{12}}},\ldots ,\;J_{\overset{ˇ}{d_{nm}}}\right)={⊕}_{j=1}^{m}\gamma_{j}\left({⊕}_{i=1}^{n}\Omega_{i}J_{\overset{ˇ}{d_{ij}}}\;\right). \end{array}$$

**Theorem 1 .**

Let ${𝔍}_{\overset{ˇ}{d_{ij}}}=\left({𝒯}_{\overset{ˇ}{d_{ij}}},\;{𝒥}_{\overset{ˇ}{d_{ij}}}\right)$ be a PFHSN, where *i* = 1,  2,…,  *n*, and *j*= 1,  2,…,  *m*. Then, using equation (13), the obtained aggregated values are also PFHSNs and

(14) $$\begin{array}{c} \text{PFHSWA }\left(J_{\overset{ˇ}{d_{11}}},\;J_{\overset{ˇ}{d_{12}}},\ldots ,\;J_{\overset{ˇ}{d_{nm}}}\right)=\langle \sqrt{1-\overset{m}{\underset{j=1}{\prod }}{\left(\overset{n}{\underset{i=1}{\prod }}{\left(1-T_{\overset{ˇ}{d_{ij}^{2}}}\right)}^{\Omega_{i}}\right)}^{\gamma_{j}}},\overset{m}{\underset{j=1}{\prod }}{\left(\overset{n}{\underset{i=1}{\prod }}{\left(J_{\overset{ˇ}{d_{ij}}}\right)}^{\Omega_{i}}\right)}^{\gamma_{j}}\rangle , \end{array}$$

where Ω_*i*_ and *γ*_*j*_ are weight vectors for experts and subattributes of the parameters.

**Proof**

Employing the mathematical induction PFHSWA operator can be proved as follows:

For *n*=1, we get Ω_1_=1. Then, we have

(15) $$\begin{array}{c} \text{PFHSWA }\left(J_{\overset{ˇ}{d_{11}}},\;J_{\overset{ˇ}{d_{12}}},\ldots ,\;J_{\overset{ˇ}{d_{nm}}}\right)={⊕}_{j=1}^{m}\gamma_{j}J_{\overset{ˇ}{d_{ij}}},\\ \text{PFHSWA }\left(J_{\overset{ˇ}{d_{11}}},\;J_{\overset{ˇ}{d_{12}}},\ldots ,\;J_{{\overset{ˇ}{d}}_{nm}}\right)=\langle \sqrt{1-\overset{m}{\underset{j=1}{\prod }}{\left(1-T_{\overset{ˇ}{d_{1j}^{2}}}\right)}^{\gamma_{j}}},{\overset{m}{\underset{j=1}{\prod }}\left(J_{\overset{ˇ}{d_{1j}}}\right)}^{\gamma_{j}}\rangle \\ =\langle \sqrt{1-\overset{m}{\underset{j=1}{\prod }}{\left(\overset{1}{\underset{i=1}{\prod }}{\left(1-T_{\overset{ˇ}{d_{1j}^{2}}}\right)}^{{Ω}_{i}}\right)}^{\gamma_{j}}},\;\overset{m}{\underset{j=1}{\prod }}{\left(\overset{1}{\underset{i=1}{\prod }}{\left(J_{\overset{ˇ}{d_{1j}}}\right)}^{{Ω}_{i}}\right)}^{\gamma_{j}}\rangle . \end{array}$$

For *m*=1, we get *γ*_1_ = 1. Then, we have

(16) $$\begin{array}{c} \text{PFHSWA }\left(J_{\overset{ˇ}{d_{11}}},\;J_{\overset{ˇ}{d_{12}}},\ldots ,\;J_{\overset{ˇ}{d_{nm}}}\right)={⊕}_{\;i=1}^{n}\Omega_{i}J_{\overset{ˇ}{d_{ij}}}\\ =\langle \sqrt{1-\overset{n}{\underset{i=1}{\prod }}{\left(1-T_{\overset{ˇ}{d_{i1}^{2}}}\right)}^{\Omega_{i}}},\;\overset{n}{\underset{i=1}{\prod }}{\left(J_{\overset{ˇ}{d_{i1}}}\right)}^{\Omega_{i}}\rangle \\ =\langle \sqrt{1-\overset{1}{\underset{j=1}{\prod }}{\left(\overset{n}{\underset{i=1}{\prod }}{\left(1-T_{\overset{ˇ}{d_{ij}^{2}}}\right)}^{{Ω}_{i}}\right)}^{\gamma_{j}}},\overset{1}{\underset{j=1}{\prod }}{\left(\overset{n}{\underset{i=1}{\prod }}{\left(J_{\overset{ˇ}{d_{ij}}}\right)}^{{Ω}_{i}}\right)}^{\gamma_{j}}\rangle . \end{array}$$

So, for *n*=1 and *m*=1, equation, (14) satisfies. Consider that equation (14) holds for *m*=*β*_1_+1, *n*=*β*_2_, *m*=*β*_1_, and *n*=*β*_2_+1, such as

(17) $$\begin{array}{c} {⊕}_{j=1}^{\beta_{1}+1}\gamma_{j}\left({⊕}_{i=1}^{\beta_{2}}{Ω}_{i}J_{\overset{ˇ}{d_{ij}}}\;\right)=\langle \sqrt{1-\overset{\beta_{1}+1}{\underset{j=1}{\prod }}{\left(\overset{\beta_{2}}{\underset{i=1}{\prod }}{\left(1-T_{\overset{ˇ}{d_{ij}^{2}}}\right)}^{{Ω}_{i}}\right)}^{\gamma_{j}}},\overset{\beta_{1}+1}{\underset{j=1}{\prod }}{\left(\overset{\beta_{2}}{\underset{i=1}{\prod }}{\left(J_{\overset{ˇ}{d_{ij}}}\right)}^{{Ω}_{i}}\right)}^{\gamma_{j}}\rangle ,\\ {⊕}_{j=1}^{\beta_{1}}\gamma_{j}\left({⊕}_{i=1}^{\beta_{2}+1}{Ω}_{i}J_{\overset{ˇ}{d_{ij}}}\right)=\langle \sqrt{1-\overset{\beta_{1}}{\underset{j=1}{\prod }}{\left(\overset{\beta_{2}+1}{\underset{i=1}{\prod }}{\left(1-T_{\overset{ˇ}{d_{ij}^{2}}}\right)}^{{Ω}_{i}}\right)}^{\gamma_{j}}},\;\overset{\beta_{1}}{\underset{j=1}{\prod }}{\left(\overset{\beta_{2}+1}{\underset{i=1}{\prod }}{\left(J_{\overset{ˇ}{d_{ij}}}\right)}^{{Ω}_{i}}\right)}^{\gamma_{j}}\rangle . \end{array}$$

For *m*=*β*_1_+1 and *n*=*β*_2_+1, we have

(18) $$\begin{array}{c} {⊕}_{j=1}^{\beta_{1}+1}\gamma_{j}\left({⊕}_{i=1}^{\beta_{2}+1}\Omega_{i}J_{\overset{ˇ}{d_{ij}}}\;\right)={⊕}_{j=1}^{\beta_{1}+1}\gamma_{j}\left({⊕}_{i=1}^{\beta_{2}}\Omega_{i}J_{\overset{ˇ}{d_{ij}}}⊕\Omega_{\beta_{2}+1}J_{\overset{ˇ}{d_{\left(\beta_{2}+1\right)j}}}\;\right)\\ ={⊕}_{j=1}^{\beta_{1}+1}{⊕}_{i=1}^{\beta_{2}}\gamma_{j}\Omega_{i}J_{\overset{ˇ}{d_{ij}}}{⊕}_{j=1}^{\beta_{1}+1}\gamma_{j}\Omega_{\beta_{2}+1}J_{\overset{ˇ}{d_{\left(\beta_{2}+1\right)j}}}\\ =\langle \begin{array}{c} \sqrt{1-\overset{\beta_{1}+1}{\underset{j=1}{\prod }}{\left(\overset{\beta_{2}}{\underset{i=1}{\prod }}{\left(1-T_{\overset{ˇ}{d_{ij}^{2}}}\right)}^{\Omega_{i}}\right)}^{\gamma_{j}}}⊕\sqrt{1-\overset{\beta_{1}+1}{\underset{j=1}{\prod }}{\left(\overset{\beta_{2}}{\underset{i=1}{\prod }}{\left(1-T_{\overset{ˇ}{d_{\left(\beta_{2}+1\right)j}^{2}}}\right)}^{\Omega_{\beta_{2}+1}}\right)}^{\gamma_{j}}}\\ \overset{\beta_{1}+1}{\underset{j=1}{\prod }}{\left(\overset{\beta_{2}}{\underset{i=1}{\prod }}{\left(J_{\overset{ˇ}{d_{ij}}}\right)}^{\Omega_{i}}\right)}^{\gamma_{j}}⊕\overset{\beta_{1}+1}{\underset{j=1}{\prod }}{\left({\left(J_{\overset{ˇ}{d_{\left(\beta_{2}+1\right)j}}}\right)}^{\Omega_{\beta_{2}+1}}\right)}^{\gamma_{j}} \end{array}\rangle \\ =\begin{array}{c} \langle \sqrt{1-\overset{\beta_{1}+1}{\underset{j=1}{\prod }}{\left(\overset{\beta_{2}+1}{\underset{i=1}{\prod }}{\left(1-T_{\overset{ˇ}{d_{ij}^{2}}}\right)}^{\Omega_{i}}\right)}^{\gamma_{j}}},\overset{\beta_{1}+1}{\underset{j=1}{\prod }}{\left(\overset{\beta_{2}+1}{\underset{i=1}{\prod }}{\left(J_{\overset{ˇ}{d_{ij}}}\right)}^{\Omega_{i}}\right)}^{\gamma_{j}}\;\rangle . \end{array} \end{array}$$

Hence, it is true for *m*=*β*_1_+1 and *n*=*β*_2_+1.

**Example 1 .**

Let *𝒰* = {*𝔲*_1_, *𝔲*_2_, *𝔲*_3_} represent the set of experts with weights Ω_*i*_ = (0.143, 0.514, 0.343)^*T*^. Experts express the beauty of a house under a defined set of attributes *ℒ*′ = {*d*_1_=lawn,  *d*_2_=security system} with their corresponding subattributes lawn = *d*_1_ = {*d*_11_=with grass,  *d*_12_=without grass} and security system =  *d*_2_ = {*d*_21_=guards,  *d*_22_= cameras}. Let *ℒ*′ = *d*_1_ × *d*_2_ be a set of subattributes:

(19) $$\begin{array}{c} L{}^{\prime }=d_{1}\times d_{2}=\left\{d_{11},\;d_{12}\right\}\times \left\{d_{21},\;d_{22}\right\}=\left\{\begin{array}{c} \left(d_{11},\;d_{21}\right),\;\left(d_{11},\;d_{22}\right),\;\left(d_{12},\;d_{21}\right),\;\left(d_{12},\;d_{22}\right) \end{array}\right\}, \end{array}$$

where $ℒ{}^{\prime }=\left\{\overset{ˇ}{d_{1}},\overset{ˇ}{d_{2}},\;\overset{ˇ}{d_{3}},\;\overset{ˇ}{d_{4}}\right\}$ represents the set of multi-subattributes with their weights *γ*_*j*_=(0.35,  0.15, 0.2, 0.3)^*T*^. Experts' opinion for each multi-subattribute in the form of PFHSNs $\left(𝔍,\;ℒ{}^{\prime }\right)={\langle {𝒯}_{\overset{ˇ}{d_{ij}}},\;{𝒥}_{\overset{ˇ}{d_{ij}}}\rangle }_{{}_{3\times 4}}$ is given as follows:

(20) $$\begin{array}{c} \left(J,L{}^{\prime }\right)=\left[\begin{array}{cccc} \left(0.3,0.8\right) & \left(0.4,0.6\right) & \left(0.3,\;0.6\right) & \left(0.5,0.6\right)\\ \left(0.8,0.3\right) & \left(0.7,0.4\right) & \left(0.7,\;0.3\right) & \left(0.4,0.8\right)\\ \left(0.3,0.6\right) & \left(0.5,0.7\right) & \left(0.6,0.5\right) & \left(0.5,0.4\right) \end{array}\right]. \end{array}$$

Using equation (14),

(21) $$\begin{array}{c} \text{PFHSWA }\left(J_{\overset{ˇ}{d_{11}}},\;J_{\overset{ˇ}{d_{12}}},\ldots ,\;J_{\overset{ˇ}{d_{nm}}}\right)=\langle \sqrt{1-\overset{4}{\underset{j=1}{\prod }}{\left(\overset{3}{\underset{i=1}{\prod }}\left({\left(1-T_{\overset{ˇ}{d_{ij}^{2}}}\right)}^{\Omega_{i}}\right)\right)}^{\gamma_{j}}},\;\overset{4}{\underset{j=1}{\prod }}{\left(\overset{3}{\underset{i=1}{\prod }}\left({\left(J_{\overset{ˇ}{d_{ij}}}\right)}^{\Omega_{i}}\right)\right)}^{\gamma_{j}}\rangle \\ =\langle \begin{array}{c} \sqrt{1-\left(\begin{array}{c} {\left\{{\left(0.91\right)}^{0.143}{\left(0.36\right)}^{0.514}{\left(0.91\right)}^{0.343}\right\}}^{0.35}{\left\{{\left(0.84\right)}^{0.143}{\left(0.51\right)}^{0.514}{\left(0.75\right)}^{0.343}\right\}}^{0.15}{\left\{{\left(0.91\right)}^{0.143}{\left(0.51\right)}^{0.514}{\left(0.64\right)}^{0.343}\right\}}^{0.2}\\ {\left\{{\left(0.84\right)}^{0.143}{\left(0.64\right)}^{0.514}{\left(0.84\right)}^{0.343}\right\}}^{0.3} \end{array}\right)}\\ \left(\begin{array}{c} {\left\{{\left(0.8\right)}^{0.143}{\left(0.3\right)}^{0.514}{\left(0.6\right)}^{0.343}\right\}}^{0.35}{\left\{{\left(0.6\right)}^{0.143}{\left(0.4\right)}^{0.514}{\left(0.7\right)}^{0.343}\right\}}^{0.15}{\left\{{\left(0.6\right)}^{0.143}{\left(0.3\right)}^{0.514}{\left(0.5\right)}^{0.343}\right\}}^{0.2}\\ {\left\{{\left(0.5\right)}^{0.143}{\left(0.4\right)}^{0.514}{\left(0.5\right)}^{0.343}\right\}}^{0.3} \end{array}\right) \end{array}\rangle \\ =\langle 0.7183\;,\;0.4839\rangle . \end{array}$$

Some properties have been presented for the PFHSWA operator based on Theorem 1.

### 3.2. Properties of PFHSWA Operator

#### 3.2.1. Idempotency

If ${𝔍}_{\overset{ˇ}{d_{ij}}}$ = ${𝔍}_{\overset{ˇ}{d}}$ = $\left({𝒯}_{\overset{ˇ}{d_{ij}}},{𝒥}_{\overset{ˇ}{d_{ij}}}\right)\forall i,j$, then $\text{PFHSWA }\left({𝔍}_{\overset{ˇ}{d_{11}}},\;{𝔍}_{\overset{ˇ}{d_{12}}},\ldots ,\;{𝔍}_{\overset{ˇ}{d_{nm}}}\right)$ =  ${𝔍}_{\overset{ˇ}{d}}$.

**Proof**

As we know that all ${𝔍}_{\overset{ˇ}{d_{ij}}}$ = ${𝔍}_{\overset{ˇ}{d}}$ = $\left({𝒯}_{\overset{ˇ}{d_{ij}}},{𝒥}_{\overset{ˇ}{d_{ij}}}\right)$, then by equation (14),

(22) $$\begin{array}{c} \text{PFHSWA }\left(J_{\overset{ˇ}{d_{11}}},\;J_{\overset{ˇ}{d_{12}}},\ldots ,\;J_{\overset{ˇ}{d_{nm}}}\right)=\langle \sqrt{1-\overset{m}{\underset{j=1}{\prod }}{\left(\overset{n}{\underset{i=1}{\prod }}\left({\left(1-T_{\overset{ˇ}{d_{ij}^{2}}}\right)}^{\Omega_{i}}\right)\right)}^{\gamma_{j}}},\overset{m}{\underset{j=1}{\prod }}{\left(\overset{n}{\underset{i=1}{\prod }}\left({\left(J_{\overset{ˇ}{d_{ij}}}\right)}^{\Omega_{i}}\right)\right)}^{\gamma_{j}}\rangle \;\\ =\langle \sqrt{1-{\left({\left(1-T_{\overset{ˇ}{d_{ij}^{2}}}\right)}^{\sum_{i=1}^{n}\Omega_{i}}\right)}^{\sum_{j=1}^{m}\gamma_{j}}},\;{\left({\left(J_{\overset{ˇ}{d_{ij}}}\right)}^{\sum_{i=1}^{n}\Omega_{i}}\right)}^{\sum_{j=1}^{m}\gamma_{j}}\rangle \\ =\langle \sqrt{1-\left(1-T_{\overset{ˇ}{d_{ij}^{2}}}\right)},\;J_{\overset{ˇ}{d_{ij}}}\rangle =\left(T_{\overset{ˇ}{d_{ij}}},J_{\overset{ˇ}{d_{ij}}}\right)=J_{\overset{ˇ}{d}}. \end{array}$$

#### 3.2.2. Boundedness

Let ${𝔍}_{\overset{ˇ}{d_{ij}}}$ be a collection of PFHSNs and ${𝔍}_{\overset{ˇ}{d_{ij}^{-}}}$ = $\langle \begin{array}{cc} \min & \min\\ j & i \end{array}\left\{{𝒯}_{\overset{ˇ}{d_{ij}}}\right\},\;\begin{array}{cc} \max & \max\\ j & i \end{array}\left\{{𝒥}_{\overset{ˇ}{d_{ij}}}\right\}\rangle$ and ${𝔍}_{\overset{ˇ}{d_{ij}^{+}}}$ = $\langle \begin{array}{cc} \max & \max\\ j & i \end{array}\left\{{𝒯}_{\overset{ˇ}{d_{ij}}}\right\},\;\begin{array}{cc} \min & \min\\ j & i \end{array}\left\{{𝒥}_{\overset{ˇ}{d_{ij}}}\right\}\rangle$; then, ${𝔍}_{\overset{ˇ}{d_{ij}^{-}}}\leq \text{PFHSWA }\left({𝔍}_{\overset{ˇ}{d_{11}}},\;{𝔍}_{\overset{ˇ}{d_{12}}},\ldots ,\;{𝔍}_{\overset{ˇ}{d_{nm}}}\right)\leq {𝔍}_{\overset{ˇ}{d_{ij}^{+}}}$.

**Proof**

As we know that ${𝔍}_{\overset{ˇ}{d_{ij}}}$ = $\left({𝒯}_{\overset{ˇ}{d_{ij}}},{𝒥}_{\overset{ˇ}{d_{ij}}}\right)$ be a PFHSN, then

(23a) $$\begin{array}{c} \underset{j}{\min}\underset{i}{\min}\left\{T_{\overset{ˇ}{d_{ij}^{2}}}\right\}\leq T_{\overset{ˇ}{d_{ij}^{2}}}\leq \underset{j}{\max}\underset{i}{\max}\left\{T_{\overset{ˇ}{d_{ij}^{2}}}\right\}\\ \Rightarrow 1-\underset{j}{\max}\underset{i}{\max}\left\{T_{\overset{ˇ}{d_{ij}^{2}}}\right\}\leq 1-T_{\overset{ˇ}{d_{ij}^{2}}}\leq 1-\underset{j}{\min}\underset{i}{\min}\left\{T_{\overset{ˇ}{d_{ij}^{2}}}\right\}\\ \Leftrightarrow {\left(1-\underset{j}{\max}\underset{i}{\max}\left\{T_{\overset{ˇ}{d_{ij}^{2}}}\right\}\right)}^{{Ω}_{i}}\leq {\left(1-T_{\overset{ˇ}{d_{ij}^{2}}}\right)}^{\Omega_{i}}\leq {\left(1-\underset{j}{\min}\underset{i}{\min}\left\{T_{\overset{ˇ}{d_{ij}^{2}}}\right\}\right)}^{\Omega_{i}}\\ \Leftrightarrow {\left(1-\underset{j}{\max}\underset{i}{\max}\left\{T_{\overset{ˇ}{d_{ij}^{2}}}\right\}\right)}^{\sum_{i=1}^{n}\Omega_{i}}\leq \overset{n}{\underset{i=1}{\prod }}{\left(1-T_{\overset{ˇ}{d_{ij}^{2}}}\right)}^{\Omega_{i}}\leq {\left(1-\underset{j}{\min}\underset{i}{\min}\left\{T_{\overset{ˇ}{d_{ij}^{2}}}\right\}\right)}^{\sum_{i=1}^{n}\Omega_{i}}\\ \Leftrightarrow {\left(1-\underset{j}{\max}\underset{i}{\max}\left\{T_{\overset{ˇ}{d_{ij}^{2}}}\right\}\right)}^{\sum_{j=1}^{m}\gamma_{j}}\leq \overset{m}{\underset{j=1}{\prod }}{\left(\overset{n}{\underset{i=1}{\prod }}{\left(1-T_{\overset{ˇ}{d_{ij}^{2}}}\right)}^{\Omega_{i}}\right)}^{\gamma_{j}}\leq {\left(1-\underset{j}{\min}\underset{i}{\min}\left\{T_{\overset{ˇ}{d_{ij}}}^{2}\right\}\right)}^{\sum_{j=1}^{m}\gamma_{j}}\\ \Leftrightarrow 1-\underset{j}{\max}\underset{i}{\max}\left\{T_{\overset{ˇ}{d_{ij}^{2}}}\right\}\leq \overset{m}{\underset{j=1}{\prod }}{\left(\overset{n}{\underset{i=1}{\prod }}{\left(1-T_{\overset{ˇ}{d_{ij}^{2}}}\right)}^{\Omega_{i}}\right)}^{\gamma_{j}}\leq 1-\underset{j}{\min}\underset{i}{\min}\left\{T_{\overset{ˇ}{d_{ij}}}^{2}\right\}\\ \Leftrightarrow \underset{j}{\min}\underset{i}{\min}\left\{T_{\overset{ˇ}{d_{ij}^{2}}}\right\}\leq 1-\overset{m}{\underset{j=1}{\prod }}{\left(\overset{n}{\underset{i=1}{\prod }}{\left(1-T_{\overset{ˇ}{d_{ij}^{2}}}\right)}^{\Omega_{i}}\right)}^{\gamma_{j}}\leq \underset{j}{\max}\underset{i}{\max}\left\{T_{\overset{ˇ}{d_{ij}}}^{2}\right\}\\ \Leftrightarrow \underset{j}{\min}\underset{i}{\min}\left\{T_{\overset{ˇ}{d_{ij}}}\right\}\leq \sqrt{1-\overset{m}{\underset{j=1}{\prod }}{\left(\overset{n}{\underset{i=1}{\prod }}{\left(1-T_{\overset{ˇ}{d_{ij}^{2}}}\right)}^{\Omega_{i}}\right)}^{\gamma_{j}}}\leq \underset{j}{\max}\underset{i}{\max}\left\{T_{\overset{ˇ}{d_{ij}}}\right\}. \end{array}$$

Similarly,

(23b) $$\begin{array}{c} \underset{j}{\min}\underset{i}{\min}\left\{J_{\overset{ˇ}{d_{ij}}}\right\}\leq \overset{m}{\underset{j=1}{\prod }}{\left(\overset{n}{\underset{i=1}{\prod }}{\left(J_{\overset{ˇ}{d_{ij}}}\right)}^{{Ω}_{i}}\right)}^{\gamma_{j}}\leq \underset{j}{\max}\underset{i}{\max}\left\{J_{\overset{ˇ}{d_{ij}}}\right\}. \end{array}$$

Let $\text{PFHSWA }\left({𝔍}_{\overset{ˇ}{d_{11}}},\;{𝔍}_{\overset{ˇ}{d_{12}}},\ldots ,\;{𝔍}_{\overset{ˇ}{d_{nm}}}\right)=\langle {𝒯}_{\overset{ˇ}{d_{\delta }}},\;{𝒥}_{\overset{ˇ}{d_{\delta }}}\rangle ={𝔍}_{\overset{ˇ}{d_{\delta }}}$; then, (23a) and (23b) inequalities can be written in the following form:

(24) $$\begin{array}{c} \underset{j}{\min}\underset{i}{\min}\left\{T_{\overset{ˇ}{d_{ij}}}\right\}\leq T_{\overset{ˇ}{d_{\delta }}}\leq \underset{j}{\max}\underset{i}{\max}\left\{T_{\overset{ˇ}{d_{ij}}}\right\}\text{ and }\underset{j}{\min}\underset{i}{\min}\left\{J_{\overset{ˇ}{d_{ij}}}\right\}\leq J_{\overset{ˇ}{d_{\delta }}}\leq \underset{j}{\max}\underset{i}{\max}\left\{J_{\overset{ˇ}{d_{ij}}}\right\}, \end{array}$$

respectively.

Using equation (11), we get

(25) $$\begin{array}{c} S\left(J_{\overset{ˇ}{d_{\delta }}}\right)=T_{\overset{ˇ}{d_{\delta }^{2}}}-J_{\overset{ˇ}{d_{\delta }^{2}}}\leq \underset{j}{\max}\underset{i}{\max}\left\{T_{\overset{ˇ}{d_{ij}}}\right\}-\underset{j}{\min}\underset{i}{\min}\left\{J_{\overset{ˇ}{d_{ij}}}\right\}=S\left(J_{\overset{ˇ}{d_{ij}^{+}}}\right),\\ S\left(J_{\overset{ˇ}{d_{\delta }}}\right)=T_{\overset{ˇ}{d_{\delta }^{2}}}-J_{\overset{ˇ}{d_{\delta }^{2}}}\geq \underset{j}{\min}\underset{i}{\min}\left\{T_{\overset{ˇ}{d_{\delta }}}\right\}-\underset{j}{\max}\underset{i}{\max}\left\{J_{\overset{ˇ}{d_{\delta }}}\right\}=S\left(J_{\overset{ˇ}{d_{ij}^{-}}}\right). \end{array}$$

Then,

(26) $$\begin{array}{c} J_{\overset{ˇ}{d_{ij}^{-}}}\leq \text{PFHSWA}\left(J_{\overset{ˇ}{d_{11}}},\;J_{\overset{ˇ}{d_{12}}},\ldots ,\;J_{\overset{ˇ}{d_{nm}}}\right)\;\leq J_{\overset{ˇ}{d_{ij}^{+}}}. \end{array}$$

#### 3.2.3. Shift Invariance

If ${𝔍}_{\overset{ˇ}{d_{\delta }}}=\langle {𝒯}_{\overset{ˇ}{d_{\delta }}},\;{𝒥}_{\overset{ˇ}{d_{\delta }}}\rangle$ be a PFHSN, then

(27) $$\begin{array}{c} \text{PFHSWA }\left(J_{\overset{ˇ}{d_{11}}}⊕J_{\overset{ˇ}{d_{\delta }}},\;J_{\overset{ˇ}{d_{12}}}⊕J_{\overset{ˇ}{d_{\delta }}},\ldots ,\;J_{\overset{ˇ}{d_{nm}}}⊕J_{\overset{ˇ}{d_{\delta }}}\right)=\text{PFHSWA }\left(J_{\overset{ˇ}{d_{11}}},\;J_{\overset{ˇ}{d_{12}}},\ldots ,\;J_{\overset{ˇ}{d_{nm}}}\right)⊕J_{\overset{ˇ}{d_{\delta }}}. \end{array}$$

**Proof**

Consider ${𝔍}_{\overset{ˇ}{d_{\delta }}}$ and ${𝔍}_{\overset{ˇ}{d_{ij}}}$ be two PFHSNs. Then, using Definition 5 (1), we have

(28) $$\begin{array}{c} J_{\overset{ˇ}{d_{\delta }}}⊕J_{\overset{ˇ}{d_{ij}}}=\langle \sqrt{T_{\overset{ˇ}{d_{\delta }^{2}}}+T_{\overset{ˇ}{d_{ij}^{2}}}-T^{2}T_{ij}^{2}},\;J_{\overset{ˇ}{d_{\delta }}}J_{\overset{ˇ}{d_{ij}}}\rangle . \end{array}$$

Therefore,

(29) $$\begin{array}{c} \text{PFHSWA }\left(J_{\overset{ˇ}{d_{11}}}⊕J_{\overset{ˇ}{d_{\delta }}},\;J_{\overset{ˇ}{d_{12}}}⊕J_{\overset{ˇ}{d_{\delta }}},\ldots ,\;J_{\overset{ˇ}{d_{nm}}}⊕J_{\overset{ˇ}{d_{\delta }}}\right)={⊕}_{\;j=1}^{m}\gamma_{j}\left({⊕}_{i=1}^{n}\Omega_{i}\left(J_{\overset{ˇ}{d_{ij}}}⊕J_{\overset{ˇ}{d_{\delta }}}\right)\;\right)\\ =\langle \sqrt{1-\overset{m}{\underset{j=1}{\prod }}{\left(\overset{n}{\underset{i=1}{\prod }}{\left(1-T_{\overset{ˇ}{d_{ij}^{2}}}\right)}^{{Ω}_{i}}\right)}^{\gamma_{j}}},\overset{m}{\underset{j=1}{\prod }}{\left(\overset{n}{\underset{i=1}{\prod }}{\left(J_{\overset{ˇ}{d_{ij}}}\right)}^{{Ω}_{i}}\right)}^{\gamma_{j}}\rangle \;\\ =\langle \sqrt{1-\left(1-T_{\overset{ˇ}{d_{\delta }^{2}}}\right)\overset{m}{\underset{j=1}{\prod }}{\left(\overset{n}{\underset{i=1}{\prod }}{\left(1-T_{\overset{ˇ}{d_{ij}^{2}}}\right)}^{{Ω}_{i}}\right)}^{\gamma_{j}}},J_{\overset{ˇ}{d_{\delta }}}\overset{m}{\underset{j=1}{\prod }}{\left(\overset{n}{\underset{i=1}{\prod }}{\left(J_{\overset{ˇ}{d_{ij}}}\right)}^{{Ω}_{i}}\right)}^{\gamma_{j}}\rangle \\ =\langle \sqrt{1-\overset{m}{\underset{j=1}{\prod }}{\left(\overset{n}{\underset{i=1}{\prod }}{\left(1-T_{\overset{ˇ}{d_{ij}^{2}}}\right)}^{{Ω}_{i}}\right)}^{\gamma_{j}}},\;\overset{m}{\underset{j=1}{\prod }}{\left(\overset{n}{\underset{i=1}{\prod }}{\left(J_{\overset{ˇ}{d_{ij}}}\right)}^{{Ω}_{i}}\right)}^{\gamma_{j}}\rangle ⊕\langle T_{\overset{ˇ}{d_{\delta }}},\;J_{\overset{ˇ}{d_{\delta }}}\rangle \\ =\text{PFHSWA }\left(J_{\overset{ˇ}{d_{11}}},\;J_{\overset{ˇ}{d_{12}}},\ldots ,\;J_{\overset{ˇ}{d_{nm}}}\right)⊕J_{\overset{ˇ}{d_{\delta }}}. \end{array}$$

#### 3.2.4. Homogeneity

Prove that $\text{PFHSWA }\left(\alpha {𝔍}_{\overset{ˇ}{d_{11}}},\;\alpha {𝔍}_{\overset{ˇ}{d_{12}}},\ldots ,\;\alpha {𝔍}_{\overset{ˇ}{d_{nm}}}\right)=\alpha \text{PFHSWA }\left({𝔍}_{\overset{ˇ}{d_{11}}},\;{𝔍}_{\overset{ˇ}{d_{12}}},\ldots ,\;{𝔍}_{\overset{ˇ}{d_{nm}}}\right)$, where *α* be a positive real number.

**Proof**

Let $\;{𝔍}_{\overset{ˇ}{d_{ij}}}$ be a PFHSN and >0; then, by using Definition 5 (3), we have

(30) $$\begin{array}{c} \alpha J_{\overset{ˇ}{d_{ij}}}=\langle \sqrt{1-{\left(1-T_{\overset{ˇ}{d_{ij}^{2}}}\right)}^{\alpha }},\;J_{\overset{ˇ}{d_{ij}^{\alpha }}}\rangle . \end{array}$$

So,

(31) $$\begin{array}{c} \text{PFHSWA }\left(\alpha J_{\overset{ˇ}{d_{11}}},\;\alpha J_{\overset{ˇ}{d_{12}}},\ldots ,\;\alpha J_{\overset{ˇ}{d_{nm}}}\right)=\langle \sqrt{1-\overset{m}{\underset{j=1}{\prod }}{\left(\overset{n}{\underset{i=1}{\prod }}{\left(1-T_{\overset{ˇ}{d_{ij}^{2}}}\right)}^{\alpha {Ω}_{i}}\right)}^{\gamma_{j}}},\;\overset{m}{\underset{j=1}{\prod }}{\left(\overset{n}{\underset{i=1}{\prod }}{\left(J_{\overset{ˇ}{d_{ij}^{2}}}\right)}^{\alpha {Ω}_{i}}\right)}^{\gamma_{j}}\rangle \\ =\langle \sqrt{1-{\left(\overset{m}{\underset{j=1}{\prod }}{\left(\overset{n}{\underset{i=1}{\prod }}{\left(1-T_{\overset{ˇ}{d_{ij}^{2}}}\right)}^{{Ω}_{i}}\right)}^{\gamma_{j}}\right)}^{\alpha }},{\left(\overset{m}{\underset{j=1}{\prod }}{\left(\overset{n}{\underset{i=1}{\prod }}{\left(J_{\overset{ˇ}{d_{ij}}}\right)}^{{Ω}_{i}}\right)}^{\gamma_{j}}\right)}^{\alpha }\rangle \\ =\alpha \text{PFHSWA }\left(J_{\overset{ˇ}{d_{11}}},\;J_{\overset{ˇ}{d_{12}}},\ldots ,\;J_{\overset{ˇ}{d_{nm}}}\right). \end{array}$$

**Definition 7 .**

Let ${𝔍}_{\overset{ˇ}{d_{ij}}}=\left({𝒯}_{\overset{ˇ}{d_{ij}}},\;{𝒥}_{\overset{ˇ}{d_{ij}}}\right)$ be a PFHSN, Ω_*i*_ and *γ*_*j*_ be weight vectors for experts and multi-subattributes of the considered attributes consistently under definite surroundings Ω_*i*_ > 0, ∑_*i*=1_^*n*^Ω_*i*_=1, *γ*_*j*_ > 0, and ∑_*j*=1_^*m*^*γ*_*j*_=1. Then, PFHSWG: Δ^*n*^⟶Δ is defined as follows:

(32) $$\begin{array}{c} \text{PFHSWG }\left(J_{\overset{ˇ}{d_{11}}},\;J_{\overset{ˇ}{d_{12}}},\ldots ,\;J_{\overset{ˇ}{d_{nm}}}\right)={⊗}_{j=1}^{m}{\left({⊗}_{i=1}^{n}J_{\overset{ˇ}{d_{nm}}}^{{Ω}_{i}}\right)}^{\gamma_{j}}. \end{array}$$

**Theorem 2 .**

Let ${𝔍}_{\overset{ˇ}{d_{ij}}}=\left({𝒯}_{\overset{ˇ}{d_{ij}}},\;{𝒥}_{\overset{ˇ}{d_{ij}}}\right)$ be a PFHSN, where *i* = 1,  2,…,  *n*,  and *j*= 1,  2,…,  *m*. Then, obtained aggregated values using equation (32) are also a PFHSN, and

(33) $$\begin{array}{c} \text{PFHSWG }\left(J_{\overset{ˇ}{d_{11}}},\;J_{\overset{ˇ}{d_{12}}},\ldots ,\;J_{\overset{ˇ}{d_{nm}}}\right)=\langle \overset{m}{\underset{j=1}{\prod }}{\left(\overset{n}{\underset{i=1}{\prod }}{\left(T_{\overset{ˇ}{d_{ij}}}\right)}^{{Ω}_{i}}\right)}^{\gamma_{j}}\;,\;\sqrt{1-\overset{m}{\underset{j=1}{\prod }}{\left(\overset{n}{\underset{i=1}{\prod }}{\left(1-J_{\overset{ˇ}{d_{ij}^{2}}}\right)}^{{Ω}_{i}}\right)}^{\gamma_{j}}}\rangle , \end{array}$$

where Ω_*i*_ and *γ*_*j*_ are weight vectors for experts and subattributes of the parameters.

**Proof**

Employing mathematical induction PFHSWG operator can be proved as follows:

For *n*=1, we get Ω_1_=1. Then, we have

(34) $$\begin{array}{c} \text{PFHSWG }\left(J_{\overset{ˇ}{d_{11}}},\;J_{\overset{ˇ}{d_{12}}},\ldots ,\;J_{\overset{ˇ}{d_{nm}}}\right)={⊗}_{j=1}^{m}J_{{\overset{ˇ}{d}}_{1j}}^{\gamma_{j}},\\ \text{PFHSWG }\left(J_{\overset{ˇ}{d_{11}}},\;J_{\overset{ˇ}{d_{12}}},\ldots ,\;J_{\overset{ˇ}{d_{nm}}}\right)=\;\langle \overset{m}{\underset{j=1}{\prod }}{\left(T_{\overset{ˇ}{d_{1j}}}\right)}^{\gamma_{j}},\;\sqrt{1-\overset{m}{\underset{j=1}{\prod }}{\left(1-J_{\overset{ˇ}{d_{1j}^{2}}}\right)}^{\gamma_{j}}}\rangle \\ =\;\langle \overset{m}{\underset{j=1}{\prod }}{\left(\overset{1}{\underset{i=1}{\prod }}{\left(T_{\overset{ˇ}{d_{ij}}}\right)}^{{Ω}_{i}}\right)}^{\gamma_{j}}\;\sqrt{1-\overset{m}{\underset{j=1}{\prod }}{\left(\overset{1}{\underset{i=1}{\prod }}{\left(1-J_{\overset{ˇ}{d_{ij}^{2}}}\right)}^{{Ω}_{i}}\right)}^{\gamma_{j}}}\rangle . \end{array}$$

For *m*=1, we have *γ*_1_ = 1. Then,

(35) $$\begin{array}{c} \text{PFHSWG }\left(J_{\overset{ˇ}{d_{11}}},\;J_{\overset{ˇ}{d_{12}}},\ldots ,\;J_{\overset{ˇ}{d_{nm}}}\right)={⊗}_{i=1}^{n}{\left(J_{\overset{ˇ}{d_{i1}}}\right)}^{{Ω}_{i}}\\ =\;\langle \overset{n}{\underset{i=1}{\prod }}{\left(T_{\overset{ˇ}{d_{i1}}}\right)}^{{Ω}_{i}},\;\sqrt{1-\overset{n}{\underset{i=1}{\prod }}{\left(1-J_{\overset{ˇ}{d_{i1}^{2}}}\right)}^{{Ω}_{i}}}\rangle \\ =\;\langle \overset{1}{\underset{j=1}{\prod }}{\left(\overset{n}{\underset{i=1}{\prod }}{\left(T_{\overset{ˇ}{d_{ij}}}\right)}^{{Ω}_{i}}\right)}^{\gamma_{j}},\;\sqrt{1-\overset{1}{\underset{j=1}{\prod }}{\left(\overset{n}{\underset{i=1}{\prod }}{\left(1-J_{\overset{ˇ}{d_{ij}^{2}}}\right)}^{{Ω}_{i}}\right)}^{\gamma_{j}}}\rangle . \end{array}$$

For *n*=1 and *m*=1, equation (33) satisfies for the PFHSWG operator. Let equation (33) hold for *m*=*β*_1_+1 and *n*=*β*_2_ and *m*=*β*_1_ and *n*=*β*_2_+1, such as

(36) $$\begin{array}{c} {⊗}_{\;j=1}^{\beta_{1}+1}{\left({⊗}_{i=1}^{\beta_{2}}{\left(J_{\overset{ˇ}{d_{ij}}}\right)}^{{Ω}_{i}}\right)}^{\gamma_{j}}=\langle \overset{\beta_{1}+1}{\underset{j=1}{\prod }}{\left(\overset{\beta_{2}}{\underset{i=1}{\prod }}{\left(T_{\overset{ˇ}{d_{ij}}}\right)}^{{Ω}_{i}}\right)}^{\gamma_{j}},\sqrt{1-\overset{\beta_{1}+1}{\underset{j=1}{\prod }}{\left(\overset{\beta_{2}}{\underset{i=1}{\prod }}{\left(1-J_{\overset{ˇ}{d_{ij}^{2}}}\right)}^{{Ω}_{i}}\right)}^{\gamma_{j}}}\rangle ,\\ {⊗}_{\;j=1}^{\beta_{1}}{\left({⊗}_{i=1}^{\beta_{2}}{\left(J_{\overset{ˇ}{d_{ij}}}\right)}^{{Ω}_{i}}\right)}^{\gamma_{j}}=\langle \overset{\beta_{1}}{\underset{j=1}{\prod }}{\left(\overset{\beta_{2}+1}{\underset{i=1}{\prod }}{\left(T_{\overset{ˇ}{d_{ij}}}\right)}^{{Ω}_{i}}\right)}^{\gamma_{j}},\sqrt{1-\overset{\beta_{1}}{\underset{j=1}{\prod }}{\left(\overset{\beta_{2}+1}{\underset{i=1}{\prod }}{\left(1-J_{\overset{ˇ}{d_{ij}^{2}}}\right)}^{{Ω}_{i}}\right)}^{\gamma_{j}}}\rangle . \end{array}$$

For *m*=*β*_1_+1 and *n*=*β*_2_+1, we have

(37) $$\begin{array}{c} {⊗}_{\;j=1}^{\beta_{1}+1}{\left({⊗}_{i=1}^{\beta_{2}+1}{\left(J_{\overset{ˇ}{d_{ij}}}\right)}^{{Ω}_{i}}\right)}^{\gamma_{j}}={⊗}_{\;j=1}^{\beta_{1}+1}{\left({⊗}_{i=1}^{\beta_{2}}{\left(J_{\overset{ˇ}{d_{ij}}}\right)}^{{Ω}_{i}}⊗{\left(J_{\overset{ˇ}{d_{\left(\beta_{2}+1\right)j}}}\right)}^{{Ω}_{\beta_{2}+1}}\;\right)}^{\gamma_{j}}\\ ={⊗}_{\;j=1}^{\beta_{1}+1}{\left({⊗}_{i=1}^{\beta_{2}}{\left(J_{\overset{ˇ}{d_{ij}}}\right)}^{{Ω}_{i}}\;\right)}^{\gamma_{j}}{⊗}_{\;j=1}^{\beta_{1}+1}{\left({\left(J_{\overset{ˇ}{d_{\left(\beta_{2}+1\right)j}^{2}}}\right)}^{{Ω}_{\beta_{2}+1}}\;\right)}^{\gamma_{j}}\\ =\langle \begin{array}{c} \overset{\beta_{1}+1}{\underset{j=1}{\prod }}{\left(\overset{\beta_{2}}{\underset{i=1}{\prod }}{\left(T_{\overset{ˇ}{d_{ij}}}\right)}^{{Ω}_{i}}\right)}^{\gamma_{j}}⊗\overset{\beta_{1}+1}{\underset{j=1}{\prod }}{\left({\left(T_{\overset{ˇ}{d_{\left(\beta_{2}+1\right)j}}}\right)}^{{Ω}_{\beta_{2}+1}}\right)}^{\gamma_{j}}\\ \sqrt{1-\overset{\beta_{1}+1}{\underset{j=1}{\prod }}{\left(\overset{\beta_{2}}{\underset{i=1}{\prod }}{\left(1-J_{\overset{ˇ}{d_{ij}^{2}}}\right)}^{{Ω}_{i}}\right)}^{\gamma_{j}}}⊗\sqrt{1-\overset{\beta_{1}+1}{\underset{j=1}{\prod }}{\left({\left(1-J_{\overset{ˇ}{d_{\left(\beta_{2}+1\right)j}^{2}}}\right)}^{{Ω}_{\beta_{2}+1}}\right)}^{\gamma_{j}}} \end{array}\rangle \\ =\langle \overset{\beta_{1}+1}{\underset{j=1}{\prod }}{\left(\overset{\beta_{2}+1}{\underset{i=1}{\prod }}{\left(T_{\overset{ˇ}{d_{ij}}}\right)}^{{Ω}_{i}}\right)}^{\gamma_{j}},\sqrt{\;1-\overset{\beta_{1}+1}{\underset{j=1}{\prod }}{\left(\overset{\beta_{2}+1}{\underset{i=1}{\prod }}{\left(1-J_{\overset{ˇ}{d_{ij}^{2}}}\right)}^{{Ω}_{i}}\right)}^{\gamma_{j}}}\rangle . \end{array}$$

Hence, it is true for *m*=*β*_1_+1 and *n*=*β*_2_+1.

**Example 2 .**

Let *𝒰* = {*𝔲*_1_, *𝔲*_2_, *𝔲*_3_} represents the set of experts with weights Ω_*i*_ =  (0.143, 0.514, 0.343)^*T*^. Experts express the beauty of a house under a defined set of attributes *ℒ*′ = {*d*_1_=lawn,  *d*_2_=security system} with their corresponding subattributes lawn = *d*_1_ = {*d*_11_=with grass,  *d*_12_=without grass} and security system = *d*_2_ = {*d*_21_=guards,  *d*_22_= cameras}. Let *ℒ*′ =  *d*_1_ × *d*_2_ be a set of subattributes:

(38) $$\begin{array}{c} L{}^{\prime }=d_{1}\times d_{2}=\left\{d_{11},\;d_{12}\right\}\times \left\{d_{21},\;d_{22}\right\}=\left\{\begin{array}{c} \left(d_{11},\;d_{21}\right),\;\left(d_{11},\;d_{22}\right),\;\left(d_{12},\;d_{21}\right),\;\left(d_{12},\;d_{22}\right) \end{array}\right\}, \end{array}$$

where *ℒ*′ = $\left\{{\overset{ˇ}{d}}_{1},{\overset{ˇ}{d}}_{2},\;{\overset{ˇ}{d}}_{3},\;{\overset{ˇ}{d}}_{4}\right\}$ represents the set of multi-subattributes with their weights *γ*_*j*_ = (0.35, 0.15, 0.2, 0.3)^*T*^. Experts' opinion for each multi-subattribute in the form of PFHSNs (*𝔍*,  *ℒ*′) = $\langle {𝒯}_{\overset{ˇ}{d_{ij}}},\;{𝒥}_{\overset{ˇ}{d_{ij}}}\rangle$ is given as follows:

(39) $$\begin{array}{c} \left(J,\;L{}^{\prime }\right)=\left[\begin{array}{cccc} \left(0.3,0.8\right) & \left(0.4,0.6\right) & \left(0.3,0.6\right) & \left(0.5,0.6\right)\\ \left(0.8,0.3\right) & \left(0.7,0.4\right) & \left(0.7,0.3\right) & \left(0.4,0.8\right)\\ \left(0.3,0.6\right) & \left(0.5,\;0.7\right) & \left(0.6,0.5\right) & \left(0.5,0.4\right) \end{array}\right]. \end{array}$$

By using equation (33),

(40) $$\begin{array}{c} \text{PFHSWG }\left(J_{\overset{ˇ}{d_{11}}},\;J_{\overset{ˇ}{d_{12}}},\ldots ,\;J_{\overset{ˇ}{d_{nm}}}\right)=\;\langle \overset{4}{\underset{j=1}{\prod }}{\left(\overset{3}{\underset{i=1}{\prod }}{\left(T_{\overset{ˇ}{d_{ij}}}\right)}^{{Ω}_{i}}\right)}^{\gamma_{j}},\;\sqrt{1-\overset{4}{\underset{j=1}{\prod }}{\left(\overset{3}{\underset{i=1}{\prod }}{\left(1-J_{\overset{ˇ}{d_{ij}^{2}}}\right)}^{{Ω}_{i}}\right)}^{\gamma_{j}}}\rangle \\ =\langle \begin{array}{c} \left(\begin{array}{c} {\left\{{\left(0.3\right)}^{0.143}{\left(0.8\right)}^{0.514}{\left(0.3\right)}^{0.343}\right\}}^{0.35}{\left\{{\left(0.4\right)}^{0.143}{\left(0.7\right)}^{0.514}{\left(0.5\right)}^{0.343}\right\}}^{0.15}{\left\{{\left(0.3\right)}^{0.143}{\left(0.7\right)}^{0.514}{\left(0.6\right)}^{0.343}\right\}}^{0.2}\\ {\left\{{\left(0.4\right)}^{0.143}{\left(0.6\right)}^{0.514}{\left(0.4\right)}^{0.343}\right\}}^{0.3} \end{array}\right),\\ \sqrt{1-\left(\begin{array}{c} {\left\{{\left(0.36\right)}^{0.143}{\left(0.91\right)}^{0.514}{\left(0.64\right)}^{0.343}\right\}}^{0.35}{\left\{{\left(0.64\right)}^{0.143}{\left(0.84\right)}^{0.514}{\left(0.51\right)}^{0.343}\right\}}^{0.15}{\left\{{\left(0.64\right)}^{0.143}{\left(0.91\right)}^{0.514}{\left(0.75\right)}^{0.343}\right\}}^{0.2}\\ {\left\{{\left(0.75\right)}^{0.143}{\left(0.84\right)}^{0.514}{\left(0.75\right)}^{0.343}\right\}}^{0.3} \end{array}\right)} \end{array}\rangle \\ =\langle 0.5646\;,\;0.5836\rangle , \end{array}$$

which established some basic properties for PFHSNs using the PFHSWG operator using Theorem 2.

### 3.3. Properties of PFHSWG Operator

#### 3.3.1. Idempotency

${𝔍}_{\overset{ˇ}{d_{ij}}}$ = ${𝔍}_{\overset{ˇ}{d}}$ = $\left({𝒯}_{\overset{ˇ}{d_{ij}}},\;{𝒥}_{\overset{ˇ}{d_{ij}}}\right)$∀*i*, *j*, then $\text{PFHSWG }\left({𝔍}_{\overset{ˇ}{d_{11}}},\;{𝔍}_{\overset{ˇ}{d_{12}}},\ldots ,\;{𝔍}_{\overset{ˇ}{d_{nm}}}\right)$ = ${𝔍}_{\overset{ˇ}{d_{\delta }}}$.

#### 3.3.2. Boundedness

Let ${𝔍}_{\overset{ˇ}{d_{ij}}}$ be a collection of PFHSNs and ${𝔍}_{\overset{ˇ}{d_{ij}^{-}}}$ = $\langle \underset{j}{\min}\underset{i}{\min}\left\{{𝒯}_{\overset{ˇ}{d_{ij}}}\right\},\;\underset{j}{\max}\underset{i}{\max}\left\{{𝒥}_{\overset{ˇ}{d_{ij}}}\right\}\rangle$ and ${𝔍}_{\overset{ˇ}{d_{ij}^{+}}}$ =  $\langle \underset{j}{\max}\underset{i}{\max}\left\{{𝒯}_{\overset{ˇ}{d_{ij}}}\right\},\;\underset{j}{\min}\underset{i}{\min}\left\{{𝒥}_{\overset{ˇ}{d_{ij}}}\right\}\rangle$; then, ${𝔍}_{\overset{ˇ}{d_{ij}^{-}}}\leq \text{PFHSWG }\left({𝔍}_{\overset{ˇ}{d_{11}}},\;{𝔍}_{\overset{ˇ}{d_{12}}},\ldots ,\;{𝔍}_{\overset{ˇ}{d_{nm}}}\right)\leq {𝔍}_{\overset{ˇ}{d_{ij}^{+}}}$.

#### 3.3.3. Shift Invariance

If ${𝔍}_{\overset{ˇ}{d_{\delta }}}=\langle {𝒯}_{\overset{ˇ}{d_{\delta }}},\;{𝒥}_{\overset{ˇ}{d_{\delta }}}\rangle$ be a PFHSN, then

(41) $$\begin{array}{c} \text{PFHSWA }\left(J_{\overset{ˇ}{d_{11}}}⊕J_{\overset{ˇ}{d_{\delta }}},\;J_{\overset{ˇ}{d_{12}}}⊕J_{\overset{ˇ}{d_{\delta }}},\ldots ,\;J_{\overset{ˇ}{d_{nm}}}⊕J_{\overset{ˇ}{d_{\delta }}}\right)=\text{PFHSWA }\left(J_{\overset{ˇ}{d_{11}}},\;J_{\overset{ˇ}{d_{12}}},\ldots ,\;J_{\overset{ˇ}{d_{nm}}}\right)⊕J_{\overset{ˇ}{d_{\delta }}}. \end{array}$$

#### 3.3.4. Homogeneity

Prove that $\text{PFHSWA }\left(\alpha {𝔍}_{\overset{ˇ}{d_{11}}},\;\alpha {𝔍}_{\overset{ˇ}{d_{12}}},\ldots ,\;\alpha {𝔍}_{\overset{ˇ}{d_{nm}}}\right)=\alpha \text{PFHSWA }\left({𝔍}_{\overset{ˇ}{d_{11}}},\;{𝔍}_{\overset{ˇ}{d_{12}}},\ldots ,\;{𝔍}_{\overset{ˇ}{d_{nm}}}\right)$ for any positive real number *α*.

## 4. Multicriteria Decision-Making Model under PFHSS Information

In the following section, we shall present the MCDM approach using the proposed PFHSWA and PFHSWG operators in the PFHSS environment.

### 4.1. Proposed Decision-Making Approach

DM is a predetermined strategy for choosing logical alternatives between multiple substances. DM blends an essential part in the factual situation. A good decision can change the course of our professional life. A sophisticated expert also analyzes the benefits and drawbacks of options then encourages a final decision. Here, we will explicate the scientific cause of the proposed approach for MCDM under the PFHSS environment. The general concept and step-by-step algorithmic rule of the projected approach are given as follows:

Consider *ℵ* =  {*ℵ*^1^,  *ℵ*^2^,  *ℵ*^3^,…,  *ℵ*^*s*^} be a set of *s* alternatives and *𝒰* = {*δ*_1_,  *δ*_2_,  *δ*_3_,…,  *δ*_*n*_} be a set of *n* experts. The weights of experts are given as Ω = (Ω_1_,  Ω_1_,…, Ω_*n*_)^*T*^ and Ω_*i*_ > 0, ∑_*i*=1_^*n*^Ω_*i*_=1. Let *𝔏* =  {*d*_1_,  *d*_2_,…,  *d*_*m*_} be expressed the set of attributes with their corresponding multi-subattributes such as *ℒ*′ = {(*d*_1*ρ*_ × *d*_2*ρ*_ × …×*d*_*mρ*_) for all *ρ* ∈ {1,  2,…,  *t*} } with weights *γ* = (*γ*_1*ρ*_,  *γ*_2*ρ*_,  *γ*_3*ρ*_,…, *γ*_*mρ*_)^*T*^ such as *γ*_*ρ*_ > 0, ∑_*ρ*=1_^*t*^*γ*_*p*_=1 = 1, and can be stated as *ℒ*′ = $\left\{\overset{ˇ}{d_{\partial }}:\partial \in \left\{1,\;2,\;\ldots ,m\right\}\right\}$. The group of experts {*κ*^*i*^: *i* = 1, 2,…, *n*} assess the alternatives {*ℵ*^(*z*)^: *z* = 1, 2,…, *s*} under the chosen subattributes {$\overset{ˇ}{d_{\partial }}$: ∂ = 1, 2,…, *k*} in the form of PFHSNs such as ${\left({ℵ}_{\overset{ˇ}{d_{ik}}}^{\left(z\right)}\right)}_{n\times m}$ = ${\left({𝒯}_{\overset{ˇ}{d_{ij}}},\;{𝒥}_{\overset{ˇ}{d_{ij}}}\right)}_{n\times m}$, where 0 ≤ ${𝒯}_{\overset{ˇ}{d_{ij}}},\;{𝒥}_{\overset{ˇ}{d_{ij}}}$ ≤ 1 and 0 ≤ ${\left({𝒯}_{\overset{ˇ}{d_{ij}}}\right)}^{2}+{\left({𝒥}_{\overset{ˇ}{d_{ij}}}\right)}^{2}\leq 1$ for all *i* and *k*. The experts provide their opinion in the form of PFHSNs ℒ_Φ_ for each alternative and present the step-wise algorithm to obtain the most suitable alternative.

Step 1. Develop decision matrices *D*^(*z*)^ = ${\left({𝒯}_{\overset{ˇ}{d_{ij}}},\;{𝒥}_{\overset{ˇ}{d_{ij}}}\right)}_{n\times m}$ in form of PFHSNs for each alternative:
  (42) $$\begin{array}{c} {\left({ℵ}^{\left(z\right)},\;L{}^{\prime }\right)}_{n\times \partial }=\begin{array}{c} \delta_{1}\\ \delta_{2}\\ \vdots \\ \delta_{n} \end{array}\left(\begin{array}{cccc} \left(T_{\overset{ˇ}{d_{11}}}^{\left(z\right)},\;{ℐ}_{\overset{ˇ}{d_{11}}}^{\left(z\right)}\right) & \left(T_{\overset{ˇ}{d_{12}}}^{\left(z\right)},\;{ℐ}_{\overset{ˇ}{d_{12}}}^{\left(z\right)}\right) & \cdots & \left(T_{\overset{ˇ}{d_{1\;\partial }}}^{\left(z\right)},\;{ℐ}_{\overset{ˇ}{d_{1\;\partial }}}^{\left(z\right)}\right)\\ \left(T_{\overset{ˇ}{d_{21}}}^{\left(z\right)},\;{ℐ}_{\overset{ˇ}{d_{21}}}^{\left(z\right)}\right) & \left(T_{\overset{ˇ}{d_{22}}}^{\left(z\right)},\;{ℐ}_{\overset{ˇ}{d_{22}}}^{\left(z\right)}\right) & \cdots & \left(T_{\overset{ˇ}{d_{2\;\partial }}}^{\left(z\right)},\;{ℐ}_{\overset{ˇ}{d_{2\;\partial }}}^{\left(z\right)}\right)\\ \vdots & \vdots & \vdots & \vdots \\ \left(T_{\overset{ˇ}{d_{n1}}}^{\left(z\right)},\;{ℐ}_{\overset{ˇ}{d_{n1}}}^{\left(z\right)}\right) & \left(T_{\overset{ˇ}{d_{n2}}}^{\left(z\right)},\;{ℐ}_{\overset{ˇ}{d_{n2}}}^{\left(z\right)}\right) & \cdots & \left(T_{\overset{ˇ}{d_{n\;\partial }}}^{\left(z\right)},\;{ℐ}_{\overset{ˇ}{d_{n\;\partial }}}^{\left(z\right)}\right) \end{array}\right). \end{array}$$
Step 2. Obtain the normalized decision matrices using normalization rules such as
  (43) $$\begin{array}{c} h_{ij}=\left\{\begin{array}{cc} J_{\overset{ˇ}{d_{ij}}}^{c}; & \text{cost type parameter,}\\ J_{\overset{ˇ}{d_{ij}}}; & \text{benefit type parameter.} \end{array}\right. \end{array}$$
 Step 3. By means of developed AOs, compute the collective decision matrix ℒ_*k*_
 Step 4. Analyze the score values to each alternate employing equation (11)
 Step 5. Indicate the premium alternate through a supreme score value ℒ_*k*_
 Step 6. Rank the alternatives

**Figure 1:**
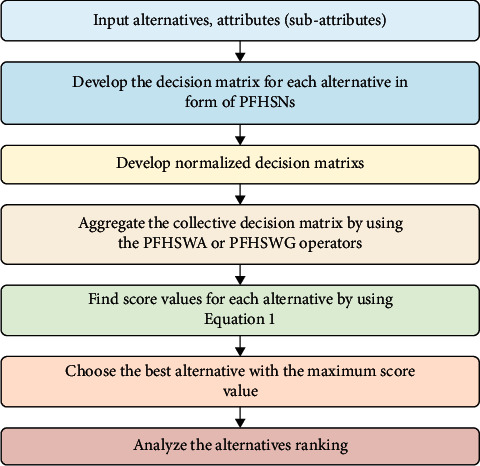
Flowchart of presented PFHSWA or PFHSWG operators.

The above-presented algorithm can be represented graphically in Figure 1.

### 4.2. Numerical Example of the Proposed MCDM Model

Let {*ℵ*^(1)^, *ℵ*^(2)^, *ℵ*^(3)^, *ℵ*^(4)^, *ℵ*^(5)^} be a set of substitutes and *𝔏* = {*d*_1_=Superiority,  *d*_2_=Delivery,  *d*_3_=Services,  *d*_4_=Troposphere,  *d*_5_=Commercial societal concern} be a collection of considered attributes given as Superiority = *d*_1_ = {*d*_11_=national level,  *d*_12_=international level}, Delivery = *d*_2_ = {*d*_21_=by carriar,  *d*_22_= by hand}, Services =  *d*_3_ = {*d*_31_=services}, Troposphere = *d*_4_ = {*d*_41_=friendly,  *d*_42_=non serious}, and Commercial societal concern = *d*_5_ = {*d*_51_=Commercial societal concern}. Let *ℒ*′ = *d*_1_ × *d*_2_ × *d*_3_ × *d*_4_ × *d*_5_ be a set of subattributes:

(44) $$\begin{array}{c} L{}^{\prime }=d_{1}\times d_{2}\times d_{3}\times d_{4}\times d_{5}=\left\{d_{11},\;d_{12}\right\}\times \left\{d_{21},\;d_{22}\right\}\times \left\{d_{31},\;d_{32}\right\}\times \left\{d_{41}\right\}\times \left\{d_{51}\right\}\\ =\left\{\begin{array}{c} \left(d_{11},\;d_{21},\;d_{31},\;d_{41},\;d_{51}\right),\;\left(d_{11},\;d_{21},\;d_{32},\;d_{41},\;d_{51}\right),\;\left(d_{11},\;d_{22},\;d_{31},\;d_{41},\;d_{51}\right),\;\left(d_{11},\;d_{22},\;d_{32},\;d_{41},\;d_{51}\right)\;\\ \left(d_{12},\;d_{21},\;d_{31},\;d_{41},\;d_{51}\right),\;\left(d_{12},\;d_{21},\;d_{32},\;d_{41},\;d_{51}\right),\;\left(d_{12},\;d_{22},\;d_{31},\;d_{41},\;d_{51}\right),\;\left(d_{12},\;d_{22},\;d_{32},\;d_{41},\;d_{51}\right)\; \end{array}\right\}, \end{array}$$

where *ℒ*′ = $\left\{\overset{ˇ}{d_{1}},\overset{ˇ}{d_{2}},\;\overset{ˇ}{d_{3}},\;\overset{ˇ}{d_{4}},\;\overset{ˇ}{d_{5}},\;\overset{ˇ}{d_{6}},\;\overset{ˇ}{d_{7}},\;\overset{ˇ}{d_{8}}\right\}$ be a set of all subattributes with weights (0.12, 0.18, 0.1, 0.15, 0.05, 0.22, 0.08, 0.1)^*T*^. Let {*u*_1_, *u*_2_, *u*_3_} be a set of three experts with weights (0.143, 0.514, 0.343)^*T*^ to judge the optimum alternative. Specialists provide their preferences in form of PFHSNs .

#### 4.2.1. By Using PFHSWA Operator

*Step* 1. The experts summarize their priorities as well as their score values in Tables 1–3 in the form of PFHSNs.
*Step* 2. No need to normalize because all attributes are the same type.
*Step* 3. By means of equation (14), specialists' judgment can be concise like this:
  (45) $$\begin{array}{c} {ℒ}_{1}=\langle \sqrt{1-\left[\begin{array}{c} {\left\{{\left(0.91\right)}^{0.143}{\left(0.51\right)}^{0.514}{\left(0.75\right)}^{0.343}\right\}}^{0.12}{\left\{{\left(0.51\right)}^{0.143}{\left(0.91\right)}^{0.514}{\left(0.36\right)}^{0.343}\right\}}^{0.18}\\ {\left\{{\left(0.64\right)}^{0.143}{\left(0.64\right)}^{0.514}{\left(0.51\right)}^{0.343}\right\}}^{0.1}{\left\{{\left(0.75\right)}^{0.143}{\left(0.91\right)}^{0.514}{\left(0.84\right)}^{0.343}\right\}}^{0.15}\\ \begin{array}{c} {\left\{{\left(0.96\right)}^{0.143}{\left(0.75\right)}^{0.514}{\left(0.84\right)}^{0.343}\right\}}^{0.05}{\left\{{\left(0.84\right)}^{0.143}{\left(0.84\right)}^{0.514}{\left(0.96\right)}^{0.343}\right\}}^{0.22}\\ {\left\{{\left(0.75\right)}^{0.143}{\left(0.51\right)}^{0.514}{\left(0.36\right)}^{0.343}\right\}}^{0.08}{\left\{{\left(0.19\right)}^{0.143}{\left(0.91\right)}^{0.514}{\left(0.51\right)}^{0.343}\right\}}^{0.1} \end{array} \end{array}\right]}\;,\left(\begin{array}{c} {\left\{{\left(0.8\right)}^{0.143}{\left(0.6\right)}^{0.514}{\left(0.7\right)}^{0.343}\right\}}^{0.12}{\left\{{\left(0.3\right)}^{0.143}{\left(0.4\right)}^{0.514}{\left(0.5\right)}^{0.343}\right\}}^{0.18}\\ {\left\{{\left(0.7\right)}^{0.143}{\left(0.5\right)}^{0.514}{\left(0.4\right)}^{0.343}\right\}}^{0.1}{\left\{{\left(0.4\right)}^{0.143}{\left(0.9\right)}^{0.514}{\left(0.3\right)}^{0.343}\right\}}^{0.15}\\ \begin{array}{c} {\left\{{\left(0.4\right)}^{0.143}{\left(0.4\right)}^{0.514}{\left(0.9\right)}^{0.343}\right\}}^{0.05}{\left\{{\left(0.6\right)}^{0.143}{\left(0.6\right)}^{0.514}{\left(0.4\right)}^{0.343}\right\}}^{0.22}\\ {\left\{{\left(0.8\right)}^{0.143}{\left(0.5\right)}^{0.514}{\left(0.4\right)}^{0.343}\right\}}^{0.08}{\left\{{\left(0.3\right)}^{0.143}{\left(0.8\right)}^{0.514}{\left(0.5\right)}^{0.343}\right\}}^{0.1} \end{array} \end{array}\right)\rangle ,\\ {ℒ}_{1}=\langle 0.5555\;,\;0.5197\rangle , \end{array}$$
  (46) $$\begin{array}{c} {ℒ}_{2}=\langle \sqrt{1-\left[\begin{array}{c} {\left\{{\left(0.64\right)}^{0.143}{\left(0.36\right)}^{0.514}{\left(0.36\right)}^{0.343}\right\}}^{0.12}{\left\{{\left(0.84\right)}^{0.143}{\left(0.51\right)}^{0.514}{\left(0.51\right)}^{0.343}\right\}}^{0.18}\\ {\left\{{\left(0.91\right)}^{0.143}{\left(0.19\right)}^{0.514}{\left(0.36\right)}^{0.343}\right\}}^{0.1}{\left\{{\left(0.19\right)}^{0.143}{\left(0.51\right)}^{0.514}{\left(0.75\right)}^{0.343}\right\}}^{0.15}\\ \begin{array}{c} {\left\{{\left(0.91\right)}^{0.143}{\left(0.84\right)}^{0.514}{\left(0.75\right)}^{0.343}\right\}}^{0.05}{\left\{{\left(0.96\right)}^{0.143}{\left(0.19\right)}^{0.514}{\left(0.51\right)}^{0.343}\right\}}^{0.22}\\ {\left\{{\left(0.51\right)}^{0.143}{\left(0.96\right)}^{0.514}{\left(0.51\right)}^{0.343}\right\}}^{0.08}{\left\{{\left(0.84\right)}^{0.143}{\left(0.91\right)}^{0.514}{\left(0.64\right)}^{0.343}\right\}}^{0.1} \end{array} \end{array}\right]}\;,\;\left(\begin{array}{c} {\left\{{\left(0.7\right)}^{0.143}{\left(0.5\right)}^{0.514}{\left(0.5\right)}^{0.343}\right\}}^{0.12}{\left\{{\left(0.6\right)}^{0.143}{\left(0.4\right)}^{0.514}{\left(0.4\right)}^{0.343}\right\}}^{0.18}\\ {\left\{{\left(0.4\right)}^{0.143}{\left(0.2\right)}^{0.514}{\left(0.5\right)}^{0.343}\right\}}^{0.1}{\left\{{\left(0.2\right)}^{0.143}{\left(0.4\right)}^{0.514}{\left(0.2\right)}^{0.343}\right\}}^{0.15}\\ \begin{array}{c} {\left\{{\left(0.8\right)}^{0.143}{\left(0.5\right)}^{0.514}{\left(0.7\right)}^{0.343}\right\}}^{0.05}{\left\{{\left(0.4\right)}^{0.143}{\left(0.3\right)}^{0.514}{\left(0.5\right)}^{0.343}\right\}}^{0.22}\\ {\left\{{\left(0.5\right)}^{0.143}{\left(0.7\right)}^{0.514}{\left(0.6\right)}^{0.343}\right\}}^{0.08}{\left\{{\left(0.5\right)}^{0.143}{\left(0.8\right)}^{0.514}{\left(0.4\right)}^{0.343}\right\}}^{0.1} \end{array} \end{array}\right)\rangle ,\\ {ℒ}_{2}=\langle 0.7252\;,\;0.4180\rangle ,\\ {ℒ}_{3}=\langle 0.5667\;,\;0.4609\rangle ,\\ {ℒ}_{4}=\langle 0.6736\;,\;0.4733\rangle ,\\ {ℒ}_{5}=\langle 0.6257,\;0.4531\rangle . \end{array}$$
  (47) $$\begin{array}{c} {ℒ}_{3}=\langle \sqrt{1-\left[\begin{array}{c} {\left\{{\left(0.51\right)}^{0.143}{\left(0.91\right)}^{0.514}{\left(0.64\right)}^{0.343}\right\}}^{0.12}{\left\{{\left(0.96\right)}^{0.143}{\left(0.84\right)}^{0.514}{\left(0.84\right)}^{0.343}\right\}}^{0.18}\\ {\left\{{\left(0.99\right)}^{0.143}{\left(0.84\right)}^{0.514}{\left(0.64\right)}^{0.343}\right\}}^{0.1}{\left\{{\left(0.91\right)}^{0.143}{\left(0.91\right)}^{0.514}{\left(0.64\right)}^{0.343}\right\}}^{0.15}\\ \begin{array}{c} {\left\{{\left(0.84\right)}^{0.143}{\left(0.64\right)}^{0.514}{\left(0.51\right)}^{0.343}\right\}}^{0.05}{\left\{{\left(0.36\right)}^{0.143}{\left(0.91\right)}^{0.514}{\left(0.36\right)}^{0.343}\right\}}^{0.22}\\ {\left\{{\left(0.64\right)}^{0.143}{\left(0.19\right)}^{0.514}{\left(0.75\right)}^{0.343}\right\}}^{0.08}{\left\{{\left(0.96\right)}^{0.143}{\left(0.51\right)}^{0.514}{\left(0.84\right)}^{0.343}\right\}}^{0.1} \end{array} \end{array}\right]}\;,\;\left(\begin{array}{c} {\left\{{\left(0.3\right)}^{0.143}{\left(0.7\right)}^{0.514}{\left(0.8\right)}^{0.343}\right\}}^{0.12}{\left\{{\left(0.5\right)}^{0.143}{\left(0.5\right)}^{0.514}{\left(0.5\right)}^{0.343}\right\}}^{0.18}\\ {\left\{{\left(0.6\right)}^{0.143}{\left(0.8\right)}^{0.514}{\left(0.5\right)}^{0.343}\right\}}^{0.1}{\left\{{\left(0.4\right)}^{0.143}{\left(0.4\right)}^{0.514}{\left(0.4\right)}^{0.343}\right\}}^{0.15}\\ \begin{array}{c} {\left\{{\left(0.6\right)}^{0.143}{\left(0.7\right)}^{0.514}{\left(0.55\right)}^{0.343}\right\}}^{0.05}{\left\{{\left(0.4\right)}^{0.143}{\left(0.4\right)}^{0.514}{\left(0.4\right)}^{0.343}\right\}}^{0.22}\\ {\left\{{\left(0.7\right)}^{0.143}{\left(0.2\right)}^{0.514}{\left(0.8\right)}^{0.343}\right\}}^{0.08}{\left\{{\left(0.5\right)}^{0.143}{\left(0.2\right)}^{0.514}{\left(0.5\right)}^{0.343}\right\}}^{0.1} \end{array} \end{array}\right)\rangle ,\\ {ℒ}_{3}=\langle 0.5667\;,\;0.4609\rangle ,\\ {ℒ}_{4}=\langle 0.6736\;,\;0.4733\rangle ,\\ {ℒ}_{5}=\langle 0.6257,\;0.4531\rangle . \end{array}$$
  (48) $$\begin{array}{c} {ℒ}_{4}=\langle \sqrt{1-\left[\begin{array}{c} {\left\{{\left(0.36\right)}^{0.143}{\left(0.75\right)}^{0.514}{\left(0.75\right)}^{0.343}\right\}}^{0.12}{\left\{{\left(0.96\right)}^{0.143}{\left(0.51\right)}^{0.514}{\left(0.19\right)}^{0.343}\right\}}^{0.18}\\ {\left\{{\left(0.96\right)}^{0.143}{\left(0.19\right)}^{0.514}{\left(0.91\right)}^{0.343}\right\}}^{0.1}{\left\{{\left(0.84\right)}^{0.143}{\left(0.36\right)}^{0.514}{\left(0.75\right)}^{0.343}\right\}}^{0.15}\\ \begin{array}{c} {\left\{{\left(0.64\right)}^{0.143}{\left(0.19\right)}^{0.514}{\left(0.91\right)}^{0.343}\right\}}^{0.05}{\left\{{\left(0.75\right)}^{0.143}{\left(0.96\right)}^{0.514}{\left(0.36\right)}^{0.343}\right\}}^{0.22}\\ {\left\{{\left(0.84\right)}^{0.143}{\left(0.84\right)}^{0.514}{\left(0.51\right)}^{0.343}\right\}}^{0.08}{\left\{{\left(0.36\right)}^{0.143}{\left(0.64\right)}^{0.514}{\left(0.96\right)}^{0.343}\right\}}^{0.1} \end{array} \end{array}\right]}\;,\left(\begin{array}{c} {\left\{{\left(0.4\right)}^{0.143}{\left(0.4\right)}^{0.514}{\left(0.7\right)}^{0.343}\right\}}^{0.12}{\left\{{\left(0.9\right)}^{0.143}{\left(0.6\right)}^{0.514}{\left(0.3\right)}^{0.343}\right\}}^{0.18}\\ {\left\{{\left(0.4\right)}^{0.143}{\left(0.3\right)}^{0.514}{\left(0.5\right)}^{0.343}\right\}}^{0.1}{\left\{{\left(0.6\right)}^{0.143}{\left(0.5\right)}^{0.514}{\left(0.7\right)}^{0.343}\right\}}^{0.15}\\ \begin{array}{c} {\left\{{\left(0.5\right)}^{0.143}{\left(0.2\right)}^{0.514}{\left(0.5\right)}^{0.343}\right\}}^{0.05}{\left\{{\left(0.6\right)}^{0.143}{\left(0.4\right)}^{0.514}{\left(0.5\right)}^{0.343}\right\}}^{0.22}\\ {\left\{{\left(0.5\right)}^{0.143}{\left(0.6\right)}^{0.514}{\left(0.5\right)}^{0.343}\right\}}^{0.08}{\left\{{\left(0.3\right)}^{0.143}{\left(0.5\right)}^{0.514}{\left(0.5\right)}^{0.343}\right\}}^{0.1} \end{array} \end{array}\right)\rangle ,\\ {ℒ}_{4}=\langle 0.6736\;,\;0.4733\rangle ,\\ {ℒ}_{5}=\langle 0.6257,\;0.4531\rangle . \end{array}$$
  (49) $$\begin{array}{c} {ℒ}_{5}=\langle \sqrt{1-\left[\begin{array}{c} {\left\{{\left(0.75\right)}^{0.143}{\left(0.36\right)}^{0.514}{\left(0.75\right)}^{0.343}\right\}}^{0.12}{\left\{{\left(0.36\right)}^{0.143}{\left(0.51\right)}^{0.514}{\left(0.84\right)}^{0.343}\right\}}^{0.18}\\ {\left\{{\left(0.51\right)}^{0.143}{\left(0.36\right)}^{0.514}{\left(0.75\right)}^{0.343}\right\}}^{0.1}{\left\{{\left(0.84\right)}^{0.143}{\left(0.75\right)}^{0.514}{\left(0.91\right)}^{0.343}\right\}}^{0.15}\\ \begin{array}{c} {\left\{{\left(0.84\right)}^{0.143}{\left(0.75\right)}^{0.514}{\left(0.51\right)}^{0.343}\right\}}^{0.05}{\left\{{\left(0.96\right)}^{0.143}{\left(0.61\right)}^{0.514}{\left(0.51\right)}^{0.343}\right\}}^{0.22}\\ {\left\{{\left(0.36\right)}^{0.143}{\left(0.51\right)}^{0.514}{\left(0.84\right)}^{0.343}\right\}}^{0.08}{\left\{{\left(0.51\right)}^{0.143}{\left(0.64\right)}^{0.514}{\left(0.75\right)}^{0.343}\right\}}^{0.1} \end{array} \end{array}\right]}\;,\;\left(\begin{array}{c} {\left\{{\left(0.7\right)}^{0.143}{\left(0.5\right)}^{0.514}{\left(0.4\right)}^{0.343}\right\}}^{0.12}{\left\{{\left(0.5\right)}^{0.143}{\left(0.4\right)}^{0.514}{\left(0.8\right)}^{0.343}\right\}}^{0.18}\\ {\left\{{\left(0.4\right)}^{0.143}{\left(0.5\right)}^{0.514}{\left(0.6\right)}^{0.343}\right\}}^{0.1}{\left\{{\left(0.3\right)}^{0.143}{\left(0.2\right)}^{0.514}{\left(0.4\right)}^{0.343}\right\}}^{0.15}\\ \begin{array}{c} {\left\{{\left(0.9\right)}^{0.143}{\left(0.7\right)}^{0.514}{\left(0.6\right)}^{0.343}\right\}}^{0.05}{\left\{{\left(0.4\right)}^{0.143}{\left(0.5\right)}^{0.514}{\left(0.5\right)}^{0.343}\right\}}^{0.22}\\ {\left\{{\left(0.4\right)}^{0.143}{\left(0.6\right)}^{0.514}{\left(0.9\right)}^{0.343}\right\}}^{0.08}{\left\{{\left(0.5\right)}^{0.143}{\left(0.4\right)}^{0.514}{\left(0.2\right)}^{0.343}\right\}}^{0.1} \end{array} \end{array}\right)\rangle ,\\ {ℒ}_{5}=\langle 0.6257,\;0.4531\rangle . \end{array}$$
*Step* 4. Utilizing equation (11), compute the score values:
  (50) $$\begin{array}{c} S\left({ℒ}_{1}\right)=\;0.03849,\\ S\left({ℒ}_{2}\right)=0.35119,\\ S\left({ℒ}_{3}\right)=0.10872,\\ S\left({ℒ}_{4}\right)=0.22972,\\ S\left({ℒ}_{5}\right)=0.18620. \end{array}$$
*Step* 5. *ℵ*^2^ has the highest score value, so *ℵ*^2^ is the premium choice.
*Step* 6. Using the considered operator, the ranking of the alternatives is given as follows: *𝕊*(ℒ_2_) > *𝕊*(ℒ_4_) > *𝕊*(ℒ_5_) > *𝕊*(ℒ_3_) > *𝕊*(ℒ_1_). So, *ℵ*^(2)^ > *ℵ*^(4)^  > *ℵ*^(5)^ > *ℵ*^(3)^>*ℵ*^(1)^.

#### 4.2.2. By Using PFHSWG Operator

*Step* 1 and *Step* 2 are similar to 4.2.1.
*Step* 3. By means of equation (33), specialists' judgment can be concise like this:
  (51) $$\begin{array}{c} {ℒ}_{1}=\langle \left(\begin{array}{c} {\left\{{\left(0.3\right)}^{0.143}{\left(0.7\right)}^{0.514}{\left(0.5\right)}^{0.343}\right\}}^{0.12}{\left\{{\left(0.7\right)}^{0.143}{\left(0.3\right)}^{0.514}{\left(0.8\right)}^{0.343}\right\}}^{0.18}\\ {\left\{{\left(0.6\right)}^{0.143}{\left(0.6\right)}^{0.514}{\left(0.7\right)}^{0.343}\right\}}^{0.1}{\left\{{\left(0.5\right)}^{0.143}{\left(0.3\right)}^{0.514}{\left(0.4\right)}^{0.343}\right\}}^{0.15}\\ \begin{array}{c} {\left\{{\left(0.2\right)}^{0.143}{\left(0.5\right)}^{0.514}{\left(0.4\right)}^{0.343}\right\}}^{0.05}{\left\{{\left(0.4\right)}^{0.143}{\left(0.4\right)}^{0.514}{\left(0.2\right)}^{0.343}\right\}}^{0.22}\\ {\left\{{\left(0.5\right)}^{0.143}{\left(0.7\right)}^{0.514}{\left(0.8\right)}^{0.343}\right\}}^{0.08}{\left\{{\left(0.9\right)}^{0.143}{\left(0.3\right)}^{0.514}{\left(0.7\right)}^{0.343}\right\}}^{0.1} \end{array} \end{array}\right)\;,\sqrt{1-\left[\begin{array}{c} {\left\{{\left(0.36\right)}^{0.143}{\left(0.64\right)}^{0.514}{\left(0.51\right)}^{0.343}\right\}}^{0.12}{\left\{{\left(0.91\right)}^{0.143}{\left(0.84\right)}^{0.514}{\left(0.75\right)}^{0.343}\right\}}^{0.18}\\ {\left\{{\left(0.51\right)}^{0.143}{\left(0.75\right)}^{0.514}{\left(0.84\right)}^{0.343}\right\}}^{0.1}{\left\{{\left(0.84\right)}^{0.143}{\left(0.19\right)}^{0.514}{\left(0.91\right)}^{0.343}\right\}}^{0.15}\\ \begin{array}{c} {\left\{{\left(0.84\right)}^{0.143}{\left(0.84\right)}^{0.514}{\left(0.19\right)}^{0.343}\right\}}^{0.05}{\left\{{\left(0.64\right)}^{0.143}{\left(0.64\right)}^{0.514}{\left(0.84\right)}^{0.343}\right\}}^{0.22}\\ {\left\{{\left(0.36\right)}^{0.143}{\left(0.75\right)}^{0.514}{\left(0.84\right)}^{0.343}\right\}}^{0.08}{\left\{{\left(0.91\right)}^{0.143}{\left(0.36\right)}^{0.514}{\left(0.75\right)}^{0.343}\right\}}^{0.1} \end{array} \end{array}\right]}\rangle ,\\ {ℒ}_{1}=\langle 0.4448,\;0.6176\rangle , \end{array}$$
  (52) $$\begin{array}{c} {ℒ}_{2}=\langle \left(\begin{array}{c} {\left\{{\left(0.6\right)}^{0.143}{\left(0.8\right)}^{0.514}{\left(0.8\right)}^{0.343}\right\}}^{0.12}{\left\{{\left(0.4\right)}^{0.143}{\left(0.7\right)}^{0.514}{\left(0.7\right)}^{0.343}\right\}}^{0.18}\\ {\left\{{\left(0.3\right)}^{0.143}{\left(0.9\right)}^{0.514}{\left(0.8\right)}^{0.343}\right\}}^{0.1}{\left\{{\left(0.9\right)}^{0.143}{\left(0.7\right)}^{0.514}{\left(0.5\right)}^{0.343}\right\}}^{0.15}\\ \begin{array}{c} {\left\{{\left(0.3\right)}^{0.143}{\left(0.4\right)}^{0.514}{\left(0.5\right)}^{0.343}\right\}}^{0.05}{\left\{{\left(0.2\right)}^{0.143}{\left(0.9\right)}^{0.514}{\left(0.7\right)}^{0.343}\right\}}^{0.22}\\ {\left\{{\left(0.7\right)}^{0.143}{\left(0.2\right)}^{0.514}{\left(0.7\right)}^{0.343}\right\}}^{0.08}{\left\{{\left(0.4\right)}^{0.143}{\left(0.3\right)}^{0.514}{\left(0.6\right)}^{0.343}\right\}}^{0.1} \end{array} \end{array}\right)\;,\sqrt{1-\left[\begin{array}{c} {\left\{{\left(0.51\right)}^{0.143}{\left(0.75\right)}^{0.514}{\left(0.75\right)}^{0.343}\right\}}^{0.12}{\left\{{\left(0.64\right)}^{0.143}{\left(0.84\right)}^{0.514}{\left(0.84\right)}^{0.343}\right\}}^{0.18}\\ {\left\{{\left(0.84\right)}^{0.143}{\left(0.96\right)}^{0.514}{\left(0.75\right)}^{0.343}\right\}}^{0.1}{\left\{{\left(0.96\right)}^{0.143}{\left(0.84\right)}^{0.514}{\left(0.96\right)}^{0.343}\right\}}^{0.15}\\ \begin{array}{c} {\left\{{\left(0.36\right)}^{0.143}{\left(0.75\right)}^{0.514}{\left(0.51\right)}^{0.343}\right\}}^{0.05}{\left\{{\left(0.84\right)}^{0.143}{\left(0.91\right)}^{0.514}{\left(0.75\right)}^{0.343}\right\}}^{0.22}\\ {\left\{{\left(0.75\right)}^{0.143}{\left(0.51\right)}^{0.514}{\left(0.64\right)}^{0.343}\right\}}^{0.08}{\left\{{\left(0.75\right)}^{0.143}{\left(0.36\right)}^{0.514}{\left(0.84\right)}^{0.343}\right\}}^{0.1} \end{array} \end{array}\right]}\rangle ,\\ {ℒ}_{2}=\langle 0.5990,0.4947\rangle ,\\ {ℒ}_{3}=\langle 0.4427,0.5516\rangle ,\\ {ℒ}_{4}=\langle 0.5021,0.5643\rangle ,\\ {ℒ}_{5}=\langle 0.5648,0.5415\rangle . \end{array}$$
  (53) $$\begin{array}{c} {ℒ}_{3}=\langle \left(\begin{array}{c} {\left\{{\left(0.7\right)}^{0.143}{\left(0.3\right)}^{0.514}{\left(0.6\right)}^{0.343}\right\}}^{0.12}{\left\{{\left(0.2\right)}^{0.143}{\left(0.4\right)}^{0.514}{\left(0.4\right)}^{0.343}\right\}}^{0.18}\\ {\left\{{\left(0.1\right)}^{0.143}{\left(0.4\right)}^{0.514}{\left(0.6\right)}^{0.343}\right\}}^{0.1}{\left\{{\left(0.3\right)}^{0.143}{\left(0.3\right)}^{0.514}{\left(0.6\right)}^{0.343}\right\}}^{0.15}\\ \begin{array}{c} {\left\{{\left(0.4\right)}^{0.143}{\left(0.6\right)}^{0.514}{\left(0.7\right)}^{0.343}\right\}}^{0.05}{\left\{{\left(0.8\right)}^{0.143}{\left(0.3\right)}^{0.514}{\left(0.8\right)}^{0.343}\right\}}^{0.22}\\ {\left\{{\left(0.6\right)}^{0.143}{\left(0.9\right)}^{0.514}{\left(0.5\right)}^{0.343}\right\}}^{0.08}{\left\{{\left(0.2\right)}^{0.143}{\left(0.7\right)}^{0.514}{\left(0.4\right)}^{0.343}\right\}}^{0.1} \end{array} \end{array}\right)\;,\sqrt{1-\left[\begin{array}{c} {\left\{{\left(0.91\right)}^{0.143}{\left(0.51\right)}^{0.514}{\left(0.36\right)}^{0.343}\right\}}^{0.12}{\left\{{\left(0.75\right)}^{0.143}{\left(0.75\right)}^{0.514}{\left(0.75\right)}^{0.343}\right\}}^{0.18}\\ {\left\{{\left(0.64\right)}^{0.143}{\left(0.36\right)}^{0.514}{\left(0.75\right)}^{0.343}\right\}}^{0.1}{\left\{{\left(0.84\right)}^{0.143}{\left(0.84\right)}^{0.514}{\left(0.84\right)}^{0.343}\right\}}^{0.15}\\ \begin{array}{c} {\left\{{\left(0.64\right)}^{0.143}{\left(0.51\right)}^{0.514}{\left(0.75\right)}^{0.343}\right\}}^{0.05}{\left\{{\left(0.84\right)}^{0.143}{\left(0.84\right)}^{0.514}{\left(0.84\right)}^{0.343}\right\}}^{0.22}\\ {\left\{{\left(0.51\right)}^{0.143}{\left(0.96\right)}^{0.514}{\left(0.36\right)}^{0.343}\right\}}^{0.08}{\left\{{\left(0.75\right)}^{0.143}{\left(0.96\right)}^{0.514}{\left(0.51\right)}^{0.343}\right\}}^{0.1} \end{array} \end{array}\right]}\rangle ,\\ {ℒ}_{3}=\langle 0.4427,0.5516\rangle ,\\ {ℒ}_{4}=\langle 0.5021,0.5643\rangle ,\\ {ℒ}_{5}=\langle 0.5648,0.5415\rangle . \end{array}$$
  (54) $$\begin{array}{c} {ℒ}_{4}=\langle \left(\begin{array}{c} {\left\{{\left(0.8\right)}^{0.143}{\left(0.5\right)}^{0.514}{\left(0.5\right)}^{0.343}\right\}}^{0.12}{\left\{{\left(0.2\right)}^{0.143}{\left(0.7\right)}^{0.514}{\left(0.9\right)}^{0.343}\right\}}^{0.18}\\ {\left\{{\left(0.2\right)}^{0.143}{\left(0.9\right)}^{0.514}{\left(0.3\right)}^{0.343}\right\}}^{0.1}{\left\{{\left(0.4\right)}^{0.143}{\left(0.8\right)}^{0.514}{\left(0.5\right)}^{0.343}\right\}}^{0.15}\\ \begin{array}{c} {\left\{{\left(0.6\right)}^{0.143}{\left(0.9\right)}^{0.514}{\left(0.3\right)}^{0.343}\right\}}^{0.05}{\left\{{\left(0.5\right)}^{0.143}{\left(0.2\right)}^{0.514}{\left(0.8\right)}^{0.343}\right\}}^{0.22}\\ {\left\{{\left(0.4\right)}^{0.143}{\left(0.4\right)}^{0.514}{\left(0.7\right)}^{0.343}\right\}}^{0.08}{\left\{{\left(0.8\right)}^{0.143}{\left(0.6\right)}^{0.514}{\left(0.2\right)}^{0.343}\right\}}^{0.1} \end{array} \end{array}\right)\;,\sqrt{1-\left[\begin{array}{c} {\left\{{\left(0.84\right)}^{0.143}{\left(0.84\right)}^{0.514}{\left(0.51\right)}^{0.343}\right\}}^{0.12}{\left\{{\left(0.19\right)}^{0.143}{\left(0.64\right)}^{0.514}{\left(0.91\right)}^{0.343}\right\}}^{0.18}\\ {\left\{{\left(0.84\right)}^{0.143}{\left(0.91\right)}^{0.514}{\left(0.75\right)}^{0.343}\right\}}^{0.1}{\left\{{\left(0.64\right)}^{0.143}{\left(0.75\right)}^{0.514}{\left(0.51\right)}^{0.343}\right\}}^{0.15}\\ \begin{array}{c} {\left\{{\left(0.75\right)}^{0.143}{\left(0.96\right)}^{0.514}{\left(0.25\right)}^{0.343}\right\}}^{0.05}{\left\{{\left(0.64\right)}^{0.143}{\left(0.84\right)}^{0.514}{\left(0.75\right)}^{0.343}\right\}}^{0.22}\\ {\left\{{\left(0.75\right)}^{0.143}{\left(0.64\right)}^{0.514}{\left(0.25\right)}^{0.343}\right\}}^{0.08}{\left\{{\left(0.91\right)}^{0.143}{\left(0.75\right)}^{0.514}{\left(0.75\right)}^{0.343}\right\}}^{0.1} \end{array} \end{array}\right]}\rangle ,\\ {ℒ}_{4}=\langle 0.5021,0.5643\rangle ,\\ {ℒ}_{5}=\langle 0.5648,0.5415\rangle . \end{array}$$
  (55) $$\begin{array}{c} {ℒ}_{5}=\langle \left(\begin{array}{c} {\left\{{\left(0.5\right)}^{0.143}{\left(0.8\right)}^{0.514}{\left(0.5\right)}^{0.343}\right\}}^{0.12}{\left\{{\left(0.8\right)}^{0.143}{\left(0.7\right)}^{0.514}{\left(0.4\right)}^{0.343}\right\}}^{0.18}\\ {\left\{{\left(0.7\right)}^{0.143}{\left(0.8\right)}^{0.514}{\left(0.5\right)}^{0.343}\right\}}^{0.1}{\left\{{\left(0.4\right)}^{0.143}{\left(0.5\right)}^{0.514}{\left(0.3\right)}^{0.343}\right\}}^{0.15}\\ \begin{array}{c} {\left\{{\left(0.4\right)}^{0.143}{\left(0.5\right)}^{0.514}{\left(0.7\right)}^{0.343}\right\}}^{0.05}{\left\{{\left(0.2\right)}^{0.143}{\left(0.7\right)}^{0.514}{\left(0.7\right)}^{0.343}\right\}}^{0.22}\\ {\left\{{\left(0.8\right)}^{0.143}{\left(0.7\right)}^{0.514}{\left(0.4\right)}^{0.343}\right\}}^{0.08}{\left\{{\left(0.7\right)}^{0.143}{\left(0.6\right)}^{0.514}{\left(0.5\right)}^{0.343}\right\}}^{0.1} \end{array} \end{array}\right)\;,\sqrt{1-\left[\begin{array}{c} {\left\{{\left(0.51\right)}^{0.143}{\left(0.75\right)}^{0.514}{\left(0.84\right)}^{0.343}\right\}}^{0.12}{\left\{{\left(0.75\right)}^{0.143}{\left(0.84\right)}^{0.514}{\left(0.36\right)}^{0.343}\right\}}^{0.18}\\ {\left\{{\left(0.84\right)}^{0.143}{\left(0.75\right)}^{0.514}{\left(0.64\right)}^{0.343}\right\}}^{0.1}{\left\{{\left(0.91\right)}^{0.143}{\left(0.96\right)}^{0.514}{\left(0.84\right)}^{0.343}\right\}}^{0.15}\\ \begin{array}{c} {\left\{{\left(0.19\right)}^{0.143}{\left(0.51\right)}^{0.514}{\left(0.64\right)}^{0.343}\right\}}^{0.05}{\left\{{\left(0.84\right)}^{0.143}{\left(0.75\right)}^{0.514}{\left(0.75\right)}^{0.343}\right\}}^{0.22}\\ {\left\{{\left(0.84\right)}^{0.143}{\left(0.64\right)}^{0.514}{\left(0.19\right)}^{0.343}\right\}}^{0.08}{\left\{{\left(0.75\right)}^{0.143}{\left(0.84\right)}^{0.514}{\left(0.96\right)}^{0.343}\right\}}^{0.1} \end{array} \end{array}\right]}\rangle ,\\ {ℒ}_{5}=\langle 0.5648,0.5415\rangle . \end{array}$$
*Step* 4. Utilizing equation (11), computes the score values:
  (56) $$\begin{array}{c} S\left({ℒ}_{1}\right)=-0.18358,\\ S\left({ℒ}_{2}\right)=0.11407,\\ S\left({ℒ}_{3}\right)=-0.10827,\\ S\left({ℒ}_{4}\right)=-0.06633,\\ S\left({ℒ}_{5}\right)=-0.02578. \end{array}$$
*Step* 5. *ℵ*^2^ has the highest score value, so *ℵ*^2^ is the premium choice.
*Step* 6. Using the considered operator, the ranking of the alternatives is given as follows: *𝕊*(ℒ_2_) > *𝕊*(ℒ_5_) > *𝕊*(ℒ_4_) > *𝕊*(ℒ_3_) > *𝕊*(ℒ_1_). So, *ℵ*^(2)^ > *ℵ*^(5)^  > *ℵ*^(4)^ > *ℵ*^(3)^>*ℵ*^(1)^.

Therefore, from the above computation, we accomplish that *ℵ*^(2)^ could be the most appropriate option.  Table 4 encompasses the whole categorization of feasible choices by PFHSWA and PFHSWG operators.

We will check that there is a dissimilarity within the evaluation results of the two operators. Such variations are due to distinctive configuration approaches. But, in both situations, the most productive and the worst correspond at most same, and this consequence summarizes the atrocity, potency, capability, as well as precision of the planned operators.

## 5. Comparative Analysis and Discussion

In the next section, we will discuss the usefulness and practicality of the projected approach with some existing techniques.

### 5.1. Advantage of the Planned Technique

Through this scientific research and communication, it is entirely convinced that the main focus of the planned approach is more general compared to the other approaches. However, the MCDM scientific method provides us additional information on the latest MCDM approach to address the hesitation in the DM process. Also, multiple mixed processes of FSS had become a unique feature of PFHSS. After including some suitable terms, as shown in Table 5, the overall details concerning the constituents may be declared correctly as well as reasonably. It could be seen that the consequences procured provide more information in comparison with existing research. Taking into consideration the multiple subattributes of the parameters, the progressed PFHSS can appropriately suppress a lot of information. Mixing inaccurate and uncertain information in the DM process is an extremely simple tool. Therefore, the projected approach is pragmatic and assorted from the existing fuzzy set hybrid structure.

### 5.2. Comparative Analysis

Two novel aggregation operators for PFHSS have been presented with their important properties and established an MCDM approach based on our developed operators. Also, we utilized our developed MCDM approach to solve decision-making complications. The results showed that the established algorithm delivers effective and precise information about alternatives comparative to existing models. The above calculation shows that *ℵ*^(2)^ is the most suitable alternative rather than other available alternatives. However, under the accessible MCDM strategies, the main advantage of the projected approach is that it provides a lot of information than the available strategies.  Table 6 below gives a comparison between the existing AOs and our advanced operators.

The available PFSWA and PFSWG [26] operators in the literature only deal with the parametrized values of the attributes of the alternatives. Sometimes, experts considered the multi-subattributes of any attribute; then, existing PFSWA and PFSWG operators cannot handle the situation. But on the contrary, our presented AOs competently deal with such limitations. Similarly, the existing PFEWA and PFEWG [38] are failed to access the parametrized values of the alternatives. Also, these operators are unable to handle the multi-subattributes of the considered parameters. The prevailing IFHSWA and IFHSWG [34] operators capably deal comparatively above-discussed operators considering the multi-subattributes. But, when the sum of Mem and Nmem values of the multi-subattributes exceeds one, then the available IFHSS cannot handle the scenario. On the contrary, our planned PFHSWA and PFHSWG operators capably accommodate the abovementioned shortcomings. Therefore, we claim forthcoming extraordinary to the existing operators we have established to be able to address the misuse as well as the obscure consequences in the overall DM procedure. Intentionally assisting with measures related to the current approach is withholding results for negative reasons. Therefore, we are sure that it is a most useful technique to evaluate inaccurate and uncertain information in the DM process.

## 6. Conclusion

In the following article, we concentrate on PFHSS to cope with unsatisfactory, fuzziness along with disparity complications by considering MD and NMD on the *n*-tuple subattributes of the considered attributes. The current scientific research encourages PFHSS operators such as PFHSWA and PFHSWG operators which were obtained by operational laws with their fundamental characteristics. Furthermore, the DM approach has been developed using PFHSWA and PFHSWG operators to deal with MCDM difficulties. Besides, comparative analysis has been carried out to confirm the effectivity and perceptibility of the projected method. Finally, based on the results procured, it could be decided that the predetermined technique deduces advanced persistency and practicability for experts in DM procedure. A subsequent study will also essence on the presentation of DM techniques using several other operators under PFHSS. Also, the developed operators can be utilized in pattern recognition, artificial intelligence, and medical diagnosis.

## Figures and Tables

**Table 1:** PFHS decision matrix for *u*_1_.

	$\overset{ˇ}{d_{1}}$	$\overset{ˇ}{d_{2}}$	$\overset{ˇ}{d_{3}}$	$\overset{ˇ}{d_{4}}$	$\overset{ˇ}{d_{5}}$	$\overset{ˇ}{d_{6}}$	$\overset{ˇ}{d_{7}}$	$\overset{ˇ}{d_{8}}$
*ℵ* ^(1)^	(0.3, 0.8)	(0.7, 0.3)	(0.6, 0.7)	(0.5, 0.4)	(0.2, 0 .4)	(0.4, 0 .6)	(0.5, 0.8)	(0.9, 0.3)
*ℵ* ^(2)^	(0.6, 0.7)	(0.4, 0 .6)	(0.3, 0.4)	(0.9, 0 .2)	(0.3, 0.8)	(0.2, 0.4)	(0.7, 0.5)	(0.4, 0.5)
*ℵ* ^(3)^	(0.7, 0.3)	(0.2, 0 .5)	(0.1, 0.6)	(0.3, 0.4)	(0.4, 0.6)	(0.8, 0.4)	(0.6, 0.7)	(0.2, 0.5)
*ℵ* ^(4)^	(0.8, 0.4)	(0.2, 0.9)	(0.2, 0.4)	(0.4, 0.6)	(0.6, 0.5)	(0.5, 0.6)	(0.4, 0.5)	(0.8, 0.3)
*ℵ* ^(5)^	(0.5, 0.7)	(0.8, 0.5)	(0.7, 0.4)	(0.4, 0.3)	(0.4, 0.9)	(0.2, 0.4)	(0.8, 0.4)	(0.7, 0.5)

**Table 2:** PFHS decision matrix for *u*_2_.

	$\overset{ˇ}{d_{1}}$	$\overset{ˇ}{d_{2}}$	$\overset{ˇ}{d_{3}}$	$\overset{ˇ}{d_{4}}$	$\overset{ˇ}{d_{5}}$	$\overset{ˇ}{d_{6}}$	$\overset{ˇ}{d_{7}}$	$\overset{ˇ}{d_{8}}$
*ℵ* ^(1)^	(0.7, 0.6)	(0.3, 0.4)	(0.6, 0.5)	(0.3, 0.9)	(0.5, 0.4)	(0.4, 0.6)	(0.7, 0.5)	(0.4, 0.8)
*ℵ* ^(2)^	(0.8, 0.5)	(0.7, 0.4)	(0.9, 0.2)	(0.7, 0.4)	(0.4, 0.5)	(0.9, 0.3)	(0.2, 0.7)	(0.3, 0.8)
*ℵ* ^(3)^	(0.3, 0.7)	(0.4, 0.5)	(0.4, 0.8)	(0.3, 0.4)	(0.6, 0.7)	(0.3, 0.4)	(0.9, 0.2)	(0.7, 0.2)
*ℵ* ^(4)^	(0.5, 0.4)	(0.7, 0.6)	(0.9, 0.3)	(0.8, 0.5)	(0.9, 0.2)	(0.2, 0.4)	(0.4, 0.6)	(0.6, 0.5)
*ℵ* ^(5)^	(0.8, 0.5)	(0.7, 0.4)	(0.8, 0.5)	(0.5, 0.2)	(0.5, 0.7)	(0.7, 0.5)	(0.7, 0.6)	(0.6, 0.4)

**Table 3:** PFHS decision matrix for *u*_3_.

	$\overset{ˇ}{d_{1}}$	$\overset{ˇ}{d_{2}}$	$\overset{ˇ}{d_{3}}$	$\overset{ˇ}{d_{4}}$	$\overset{ˇ}{d_{5}}$	$\overset{ˇ}{d_{6}}$	$\overset{ˇ}{d_{7}}$	$\overset{ˇ}{d_{8}}$
*ℵ* ^(1)^	(0.5, 0.7)	(0.8, 0.5)	(0.7, 0.4)	(0.4, 0.3)	(0.4, 0.9)	(0.2, 0.4)	(0.8, 0.4)	(0.7, 0.5)
*ℵ* ^(2)^	(0.8, 0.5)	(0.7, 0.4)	(0.8, 0.5)	(0.5, 0.2)	(0.5, 0.7)	(0.7, 0.5)	(0.7, 0.6)	(0.6, 0.4)
*ℵ* ^(3)^	(0.6, 0.8)	(0.4, 0.5)	(0.6, 0.5)	(0.6, 0.4)	(0.7, 0.5)	(0.8, 0.4)	(0.5, 0.8)	(0.4, 0.5)
*ℵ* ^(4)^	(0.5, 0.7)	(0.9, 0.3)	(0.3, 0.5)	(0.5, 0.7)	(0.3, 0.5)	(0.8, 0.5)	(0.7, 0.5)	(0.2, 0.5)
*ℵ* ^(5)^	(0.5, 0.4)	(0.4, 0.8)	(0.5, 0.6)	(0.3, 0.4)	(0.7, 0.6)	(0.7, 0.5)	(0.4, 0.9)	(0.5, 0.2)

**Table 4:** Alternatives score values with their ranking.

Method	*ℵ* ^(1)^	*ℵ* ^(2)^	*ℵ* ^(3)^	*ℵ* ^(4)^	*ℵ* ^(5)^	Alternatives ranking
PFHSWA operator	0.03849	0.35119	0.10872	0.22972	0.18620	*ℵ*^(2)^ > *ℵ*^(4)^ > *ℵ*^(5)^ > *ℵ*^(3)^ > *ℵ*^(1)^
PFHSWG operator	−0.18358	0.11407	−0.10827	−0.06633	−0.02578	*ℵ*^(2)^ > *ℵ*^(5)^ > *ℵ*^(4)^ > *ℵ*^(3)^ > *ℵ*^(1)^

**Table 5:** Comparison of PFHSSs with some prevailing models.

	Set	Truthiness	Falsity	Parametrization	Attributes	Subattributes	Limitations
Zadeh [1]	FS	✓	×	×	✓	×	Unable to handle the NMD of multi-subattributes
Maji et al. [36]	FSS	✓	×	✓	×	×	Deals with the parametrization of the alternatives but is unable to handle the NMD of multi-subattributes
Atanassov [2]	IFS	✓	✓	×	✓	×	Unable to handle the multi-subattributes of the parameters
Maji et al. [37]	IFSS	✓	✓	✓	✓	×	Cannot deal with problems that satisfy 1 < MD + NMD + ≤ + 0
Peng et al. [21]	PFSS	✓	✓	✓	✓	×	Cannot deal with problems that satisfy 1 < MD^2^ + NMD^2^ + ≤ + 0
Zulqarnain et al. [33]	IFHSS	✓	✓	✓	✓	✓	Cannot deal with problems multi-subattributes 1 + < + MD + NMD + ≤ + 0
Proposed approach	PFHSS	✓	✓	✓	✓	✓	Cannot deal with problems in which multi-subattributes of parameters satisfy 1 < MD^2^ + NMD^2^ ≤ 0

**Table 6:** Comparative analysis with existing operators.

Method	Score values for alternatives					Ranking order
	*ℵ* ^(1)^	*ℵ* ^(2)^	*ℵ* ^(3)^	*ℵ* ^(4)^	*ℵ* ^(5)^	
PFSWA [23]	0.21173	0.33215	0.22017	0.27008	0.21893	*ℵ*^(2)^ > *ℵ*^(4)^ > *ℵ*^(3)^ > *ℵ*^(5)^ > *ℵ*^(1)^
PFSWG [23]	0.20587	0.32902	0.23066	0.25462	0.21727	*ℵ*^(2)^ > *ℵ*^(4)^ > *ℵ*^(3)^ > *ℵ*^(5)^ > *ℵ*^(1)^
PFEWA [38]	0.51686	0.60467	0.54833	0.59021	0.51235	*ℵ*^(2)^ > *ℵ*^(4)^ > *ℵ*^(3)^ > *ℵ*^(1)^ > *ℵ*^(5)^
PFEWG [38]	0.54219	0.62190	0.56597	0.59381	0.52209	*ℵ*^(2)^ > *ℵ*^(4)^ > *ℵ*^(3)^ > *ℵ*^(1)^ > *ℵ*^(5)^
IFHSWA [34]	0.41735	0.49830	0.46175	0.43247	0.40935	*ℵ*^(2)^ > *ℵ*^(3)^ > *ℵ*^(4)^ > *ℵ*^(1)^ > *ℵ*^(5)^
IFHSWG [34]	0.36175	0.42615	0.40790	0.40635	0.35635	*ℵ*^(2)^ > *ℵ*^(3)^ > *ℵ*^(4)^ > *ℵ*^(1)^ > *ℵ*^(5)^
PFHSWA operator	0.03849	0.35119	0.10872	0.22972	0.18620	*ℵ*^(2)^ > *ℵ*^(4)^ > *ℵ*^(5)^ > *ℵ*^(3)^ > *ℵ*^(1)^
PFHSWG operator	−0.18358	0.11407	−0.10827	−0.06633	−0.02578	*ℵ*^(2)^ > *ℵ*^(5)^ > *ℵ*^(4)^ > *ℵ*^(3)^ > *ℵ*^(1)^

## Data Availability

No data were used to support the findings of the study.

## References

[1] Zadeh L. A. (1965). Fuzzy sets. *Information and Control*.

[2] Atanassov K. T. (1986). Intuitionistic fuzzy sets. *Fuzzy Sets and Systems*.

[3] Yager R. R. Pythagorean fuzzy subsets.

[4] Yager R. R. (2014). Pythagorean membership grades in multicriteria decision making. *IEEE Transactions on Fuzzy Systems*.

[5] Zhang X., Xu Z. (2014). Extension of TOPSIS to multiple criteria decision making with pythagorean fuzzy sets. *International Journal of Intelligent Systems*.

[6] Wang L., Li N. (2020). Pythagorean fuzzy interaction power bonferroni mean aggregation operators in multiple attribute decision making. *International Journal of Intelligent Systems*.

[7] Gao H., Lu M., Wei G., Wei Y. (2018). Some novel pythagorean fuzzy interaction aggregation operators in multiple attribute decision making. *Fundamenta Informaticae*.

[8] Wei G. (2017). Pythagorean fuzzy interaction aggregation operators and their application to multiple attribute decision making. *Journal of Intelligent & Fuzzy Systems*.

[9] Talukdar P., Goala S., Dutta P., Limboo B. (2020). Fuzzy multicriteria decision making in medical diagnosis using an advanced distance measure on linguistic Pythagorean fuzzy sets. *Annals of Optimization Theory and Practice*.

[10] Wang L., Garg H., Li N. (2020). Pythagorean fuzzy interactive hamacher power aggregation operators for assessment of express service quality with entropy weight. *Soft Computing*.

[11] Ejegwa P. A., Onyeke I. C., Adah V. (2020). An algorithm for an improved intuitionistic fuzzy correlation measure with medical diagnostic application. *Annals of Optimization Theory and Practice*.

[12] Peng X., Yang Y. (2015). Some results for pythagorean fuzzy sets. *International Journal of Intelligent Systems*.

[13] Garg H. (2019). New logarithmic operational laws and their aggregation operators for pythagorean fuzzy set and their applications. *International Journal of Intelligent Systems*.

[14] Arora R., Garg H. (2019). Group decision-making method based on prioritized linguistic intuitionistic fuzzy aggregation operators and its fundamental properties. *Computational and Applied Mathematics*.

[15] Ma Z., Xu Z. (2016). Symmetric pythagorean fuzzy weighted geometric/averaging operators and their application in multicriteria decision-making problems. *International Journal of Intelligent Systems*.

[16] Molodtsov D. (1999). Soft set theory-First results. *Computers & Mathematics with Applications*.

[17] Maji P. K., Biswas R., Roy A. R. (2003). Soft set theory. *Computers and Mathematics with Applications*.

[18] Garg H., Arora R. (2018). Generalized and group-based generalized intuitionistic fuzzy soft sets with applications in decision-making. *Applied Intelligence*.

[19] Garg H., Arora R., Arora R. (2020). TOPSIS method based on correlation coefficient for solving decision-making problems with intuitionistic fuzzy soft set information. *AIMS Mathematics*.

[20] Zulqarnain R. M., Xin X. L., Saqlain M., Khan W. A. (2021). TOPSIS method based on the correlation coefficient of interval-valued intuitionistic fuzzy soft sets and aggregation operators with their application in decision-making. *Journal of Mathematics*.

[21] Peng X., Yang Y., Song J. (2015). Pythagoren fuzzy soft set and its application. *Computer Engineering*.

[22] Athira T. M., John S. J., Garg H. (2019). Entropy and distance measures of pythagorean fuzzy soft sets and their applications. *Journal of Intelligent & Fuzzy Systems*.

[23] Zulqarnain R. M., Xin X. L., Garg H., Khan W. A. (2021). Aggregation operators of pythagorean fuzzy soft sets with their application for green supplier chain management. *Journal of Intelligent & Fuzzy Systems*.

[24] Riaz M., Naeem K., Afzal D. (2020). Pythagorean m-polar fuzzy soft sets with TOPSIS method for MCGDM. *Punjab University Journal of Mathematics*.

[25] Zulqarnain R. M., Xin X. L., Garg H., Ali R. (2021). Interaction aggregation operators to solve multi criteria decision making problem under pythagorean fuzzy soft environment. *Journal of Intelligent & Fuzzy Systems*.

[26] Riaz M., Khalid N., Afzal D. (2020). A similarity measure under pythagorean fuzzy soft environment with applications. *Computational and Applied Mathematics*.

[27] Zulqarnain R. M., Xin X. L., Siddique I., Asghar Khan W., Yousif M. A. (2021). TOPSIS method based on correlation coefficient under pythagorean fuzzy soft environment and its application towards green supply chain management. *Sustainability*.

[28] Smarandache F. (2018). Extension of soft set to hypersoft set, and then to plithogenic hypersoft set. *Neutrosophic Sets and Systems*.

[29] Zulqarnain R. M., Xin X. L., Saqlain M., Smarandache F. (2020). Generalized aggregate operators on neutrosophic hypersoft set. *Neutrosophic Sets and Systems*.

[30] Zulqarnain R. M., Xin X. L., Saeed M. (2021). *A Development of Pythagorean Fuzzy Hypersoft Set with Basic Operations and Decision-Making Approach Based on the Correlation Coefficient, Theory and Application of Hypersoft Set*.

[31] Samad A., Zulqarnain R. M., Sermutlu E. (2021). Selection of an effective hand sanitizer to reduce COVID-19 effects and extension of TOPSIS technique based on correlation coefficient under neutrosophic hypersoft set. *Complexity*.

[32] Zulqarnain R. M., Siddique I., Ali R., Jarad F., Samad A., Abdeljawad T. (2021). Neutrosophic hypersoft matrices with application to solve multiattributive decision-making problems. *Complexity*.

[33] Zulqarnain R. M., Xin X. L., Saeed M. (2020). Extension of TOPSIS method under intuitionistic fuzzy hypersoft environment based on correlation coefficient and aggregation operators to solve decision making problem. *AIMS Mathematics*.

[34] Zulqarnain R. M., Siddique I., Ali R., Pamucar D., Marinkovic D., Bozanic D. (2021). Robust aggregation operators for intuitionistic fuzzy hypersoft set with their application to solve MCDM problem. *Entropy*.

[35] Zulqarnain R. M., Saddique I., Jarad F., Ali R., Abdeljawad T. (2021). Development of TOPSIS technique under pythagorean fuzzy hypersoft environment based on correlation coefficient and its application towards the selection of antivirus mask in COVID-19 pandemic. *Complexity*.

[36] Maji P. K., Biswas R., Roy A. R. (2001). Fuzzy soft sets. *Journal of Fuzzy Mathematics*.

[37] Maji P. K., Biswas R., Roy A. R. (2001). Intuitionistic fuzzy soft sets. *Journal of Fuzzy Mathematics*.

[38] Garg H. (2016). A new generalized Pythagorean fuzzy information aggregation using Einstein operations and its application to decision making. *International Journal of Intelligent Systems*.

